# Functional characterization of soybean strigolactone biosynthesis and signaling genes in Arabidopsis MAX mutants and GmMAX3 in soybean nodulation

**DOI:** 10.1186/s12870-017-1182-4

**Published:** 2017-12-21

**Authors:** Basir UI Haq, Muhammad Zulfiqar Ahmad, Naveed ur Rehman, Junjie Wang, Penghui Li, Dongqin Li, Jian Zhao

**Affiliations:** 1 National Key Laboratory of Crop Genetic Improvement, Huazhong Agricultural University, Wuhan, 430075 China; 2 State Key Laboratory of Tea Plant Biology and Utilization, College of Tea and Food Science and Technology, Anhui Agricultural University, Hefei, 230036 China

**Keywords:** Soybean, Strigolactones, Nodulation, Hormone interaction, GmMAX3, Genetic complementation

## Abstract

**Background:**

Strigolactones (SLs) play important roles in controlling root growth, shoot branching, and plant-symbionts interaction. Despite the importance, the components of SL biosynthesis and signaling have not been unequivocally explored in soybean.

**Results:**

Here we identified the putative components of SL synthetic enzymes and signaling proteins in soybean genome. Soybean genome contains conserved MORE AXILLARY BRANCHING (MAX) orthologs, GmMAX1s, GmMAX2s, GmMAX3s, and GmMAX4s. The tissue expression patterns are coincident with SL synthesis in roots and signaling in other tissues under normal conditions. *GmMAX1a*, *GmMAX2a*, *GmMAX3b*, and *GmMAX4a* expression in their Arabidopsis orthologs’ mutants not only restored most characteristic phenotypes, such as shoot branching and shoot height, leaf shape, primary root length, and root hair growth, but also restored the significantly changed hormone contents, such as reduced JA and ABA contents in all mutant leaves, but increased auxin levels in *atmax1, atmax3* and *atmax4* mutants. Overexpression of these *GmMAX*s also altered the hormone contents in wild-type Arabidopsis. GmMAX3b was further characterized in soybean nodulation with overexpression and knockdown transgenic hairy roots. *GmMAX3b* overexpression (*GmMAX3b*-*OE*) lines exhibited increased nodule number while *GmMAX3b* knockdown (*GmMAX3b-KD*) decreased the nodule number in transgenic hairy roots. The expression levels of several key nodulation genes were also altered in GmMAX3b transgenic hairy roots. *GmMAX3b* overexpression hairy roots had reduced ABA, but increased JA levels, with no significantly changed auxin content, while the contrast changes were observed in *GmMAX3b-KD* lines. Global gene expression in *GmMAX3b-OE* or *GmMAX3b-KD* hairy roots also revealed that altered expression of *GmMAX3b* in soybean hairy roots changed several subsets of genes involved in hormone biosynthesis and signaling and transcriptional regulation of nodulation processes.

**Conclusions:**

This study not only revealed the conservation of SL biosynthesis and signaling in soybean, but also showed possible interactions between SL and other hormone synthesis and signaling during controlling plant development and soybean nodulation. GmMAX3b-mediated SL biosynthesis and signaling may be involved in soybean nodulation by affecting both root hair formation and its interaction with rhizobia.

**Electronic supplementary material:**

The online version of this article (10.1186/s12870-017-1182-4) contains supplementary material, which is available to authorized users.

## Background

The currently emerging hormones, the strigolactone (SLs), a novel group of terpeniod lactone derived from carotenoid, were first recognized as a constituent of root secretion for germination of parasitic witch weeds [71]. SLs then are found to be required for the establishment of symbiotic arbuscular mycorrhizal fungi in plant related to phosphor deficiency [3, 12, 63], and recently involved in legume-rhizobia interaction [22–24, 37]. The most prominent phenotypes controlled by SLs are the root growth, shoot branching, and overall plant architecture [25, 54, 55, 60].

Genetic and physiological studies on the carotenoid –derived long-distance signal molecules had revealed that SLs are mainly produced in the roots of plants and then transported upward to shoot regions [25, 60]. These studies have revealed the critical roles of SLs in controlling shoot branching in multi-branching mutants, including MORE AXILLARY GROWTH (MAX) in Arabidopsis, RAMOSUS (RMS) in pea (*Pisum sativum*), DECREASED APICAL DOMINANCE (DAD) in petunia (*Petunia hybrida*), and DWARF or HIGH-TILLERING DWARF (D/HTD) in rice (*Oryza sativa*) [5, 9, 11, 25, 60]. SLs also have roles in plant development and adaptive responses other than regulating shoot branching [16, 49]. It has been demonstrated that these MAX/D/RMS/DAD mutants are involved in either SL biosynthesis or SL signal perception and transductions [6, 7]. SL biosynthesis was derived from carotenoid pathway, firstly by D27 carotenoid isomerase catalyzed conversion of all-trans-β-carotene into produce 9-*cis*-β-carotene [4, 68]. 9-Cis-β-carotene is successively cleaved by the carotenoid cleavage dioxygenase 7 (CCD7), encoded by Arabidopsis AtMAX3, rice D17/HTD1, pea RMS5, or petunina DAD3 to produce 9-*cis*-β-apo-10-carotenal [4] and CCD8, encoded by AtMAX4, rice D10, pea RMS1 or petunia DAD1, to convert 9-*cis*-β-apo-10 carotenal into carlactone [4]. Carlactone can be further converted into 5-deoxylstrigol and other bioactive SLs by a P450 monooxygenase, encoded by Arabidopsis MAX1 and lotus LBO [1, 15, 48, 74]. These bioactive SLs are perceived by rice D14 or petunia DAD2, an α/β-fold hydrolase that can hydrolyze SLs, acting as a SL receptor. D14 interacts with the *MAX2/D3* group of F-box proteins [18, 20, 26, 55, 72] to form a D14/ SKP1–CULLIN–F-BOX (SCF) E3 ubiquitin ligase complex D14-SCF^D3/MAX2^ in the presence of SLs [55]. The D14-SCF^D3/MAX2^ appears to play a vital role in mediating SLs-triggered its substrate protein degradation [18, 55, 68, 75]. D53 in rice or its orthologs, SMAX1-LIKE6 (SMXL6) and SMXL7 in Arabidopsis act as a substrate for SCF^D3/At*MAX2*^ [29, 36, 65, 75]. As a repressor, D53/SMXL6/7 degradation by SCF^D3/At*MAX2*^ ubiquitination complex and 26S proteosome then release the suppressed SL signaling pathways, even downstream targets of SL signaling remain to be disclosed [6, 13, 53]. Since SLs are found synthesized in roots and stems, then transported upwards to shoot through hypodermal passage cells instead of xylem in higher plant parts [32, 70, 73], an ATP-binding cassette (ABC) transporter PLEIOTROPIC DRUG RESISTANCE1 (PDR1) was identified as a SL exporter [33, 46]. Although great progress has been made in understanding SL biosynthesis and signaling, more essential details and underlying mechanisms underlying of many SLs-related phenomena, e.g. complex cross-talks or interactions between SLs and other hormones, remain to be determined.

Functions of SLs in plants are mostly through complex interactions with other hormones such as auxins, cytokinins, abscisic acid (ABA), jasmonate acid (JA) and oxylipins, and gibberellic acids (GAs). SLs and auxins together control shoot budding and branching [17, 50]. SLs and JA interaction in Arbuscular mycorrhizal colonization [40], SLs influencing root development through the cytokinin signaling network or interaction with ethylene and auxin [29, 31], gibberellin signaling regulating SL biosynthesis [27]. Among them, the interactions between SL and auxin biosynthesis, transport, and signaling are extensively studied [17, 19]. However, due to the complex and wide-effects of these interactions, mechanisms underlying the cross-talking hormones and relevant developmental or stress responsive phenotypes yet to be understood.

Despite of the important roles of SLs in controlling shoot and root architectures demonstrated in different model plants species such as Arabidopsis, rice, pea and petunia, and SLs are directly related to agronomic traits for many crops, the relevant parts in several important leguminous crops such as soybean and alfalfa remain to be explored. Current studies have revealed that diverse and parallel strigolactone biosynthesis pathways and signal transduction mechanisms could exist in different plants species [6]. It is of great interest and importance to investigate how strigolactones are synthesized, how their signals are transduced and function in soybean. Here we identified the putative orthologs of Arabidopsis MAX1, 2, 3, and 4 from soybean genome, based on homology to their counterparts in these model plants and the expression patterns. We analyzed their functions by genetic complementation of Arabidopsis corresponding mutants, with regarding to several developmental phenotypes. We further characterized GmMAX3b for its function in nodulation process, using transgenic hairy roots in combination of transcriptomic analysis. The study provided insights into our understanding of SL function in soybean-rhizobia interaction. We investigated the physiological functions of GmMAX3b by overexpression and knockdown in soybean transgenic hairy roots for their nodulation phenotype, hormone content, nodulation gene expression changes. *GmMAX3b-OE* chimerical transgenic plant hairy roots displayed more nodules while *GmMAX3b*-*KD* plants gave less nodules than the control hairy roots did. Expression of several key nodulation genes were changed correspondingly, and contents of hormones like IAA, ABA, and JA also altered in transgenic hairy roots. All these data suggest that GmMAX3b not only plays a conserved role in regulating shoot branching, and root developments, like its ortholog, but also functions in root hair formation and nodulation in soybean.

## Methods

### Plant materials and growth conditions

*Arabidopsis thaliana* wild-type (ecotype Columbia-0, Col-0) and *atmax* mutants, *atmax1–1* (SAIL_25_A05, ABRC stock #: CS9564), *atmax2–1* (SALK_028336, ABRC stock #: CS9566, #: CS9565), *atmax3–9* (ABRC stock #: CS9567), *atmax4–1*(ABRC stock #: CS9568) were obtained from the Arabidopsis Biological Resources Center (ABRC, Columbus, Ohio, USA). Arabidopsis *atmax2–1, atmax3* mutant has been described previously [35, 54]. All seeds were surface-sterilized and subsequently germinated on one-half strength Murashige and Skoog (MS) agar plates supplemented with 1% (*w*/*v*) sucrose in 12 h/12 h light/dark photoperiod at day/night temperatures of 23 °C/20 °C and 400 μmol m^−2^ s^−1^ with a 16-h photoperiod. 10 day-old Arabidopsis seedlings were transferred from MS medium to soil pots grown in growth chambers at 23 °C, approximately 125 μmol photons m^−2^ s^−1^ with 14 h/10 h (long-day conditions) or 10 h/14 h (short-day conditions) photoperiods, according to experiment requirements.

### Gene cloning and vector construction

Homologues of Arabidopsis MAX genes and rice DWARF genes were identified through homology search against soybean genome on public database (Phytozome.org). For gene cloning, total RNA was extracted from 14-days old *Glycine max* leaves, stems, or roots of soybean (*Glycine max*) variety “Tianlong #1”. About 10 μg of total RNA was used to synthesize first-strand cDNA using the first-strand synthesis system (Invitrogen). The cDNA were used as a template for amplification of the open reading frames (ORFs) of *GmMAX* genes with pairs of gene-specific primers (Additional file 1: Table S1). After all *GmMAX* ORFs were cloned into T-easy vector and sequenced for verification, the ORFs for *GmMAX1a* (Glyma04g05510.3) *GmMAX1b (*Glyma06g05520.2), *GmMAX2a* (Glyma.12 g15360.1), *GmMAX3b* (Glyma11g16370.1), *GmMAX4a (*Glyma04g08910.1**),** *GmMAX4b (*Glyma06g09000.2), were amplified by using long primers for being subcloned into pDONR221 by using recombination enzyme BP clonase (Life Technologies). The resulted pDONR221 clones harboring various *GmMAX* ORFs were verified by sequencing and then recombined into different Gateway destination vectors, including pB2GW7 for overexpression and pB7GWIWGII for knockdown using LR clonase (Life technologies, CA, USA).

### Arabidopsis transformation and mutant complementation

pB2GW7 binary vectors harboring 35S::*GmMAX1a,* 35S::*GmMAX1b,* 35S::*GmMAX2a,* 35S::*GmMAX3b,* 35S::*GmMAX4a,* or 35S::*GmMAX4b,* were transformed into *Agrobacterium tumefaciens* GV3101 by electroporation. Selected *A. tumefaciens* GV3101 clones were grown overnight for Arabidopsis transformation using flower dipping method. For overexpression of *GmMAX* genes in *Arabidopsis thaliana*, Col-0 plants were used for transformation. For *max* mutant complementation, *at* *max1, atmax2, atmax3,* and *atmax4* mutant plants were transformed with *A. tumefaciens* GV3101 harboring each corresponding *GmMAX* homologue gene. All transformants were screened and selected by using BASTA spraying, and at least 10 independent homozygous T3 transgenic lines were selected for our phenotype observation. Three independent transgenic lines were used for further analysis.

When plants reached maturity, the number of primary rosette leaf branches was counted. A minimum of 10 individual plants per genotype were examined. Expression of the transgene in transformants was confirmed by qRT-PCR. T3 Arabidopsis transgenic plants were measured for branching inhibition and analysis of plants hormones.

### Evaluation of branches number, leaf size, and leaf shape

Branches number and leaf development analyses were performed on soil-grown plants, according to the method described previously [50, 54]. The seeds of various genotypes were sown  on soil pots, and Arabidopsis seedlings  were separated each to one pot at 7 days after sowing. Plants were grown under continuous light (4 μmol/m^2^/s) at 22 °C. The number of primary rosette branches was counted and shoot height was measured at 56 days after sowing. For each genotype, 21-day-old plants were photographed for determination of leaf size. The leaf area was then measured using the Image-J software package. For evaluation of leaf shape, the leaves were removed from plants after 35-day-old plants and pictured for measurement of leaf length and width ration using the same software.

### Analysis of hypocotyl length, primary root and root hair length

The hypocotyl length, primary root length, and root hair density and length in Arabidopsis roots and soybean hairy roots were assessed by using methods described previously [30] with little modifications. Briefly, plates containing Arabidopsis seeds were germinated and incubated vertically under continuous light. Observation and measurements were conducted at 8–10 days after sowing, by photographing and measurement using the Image-J software NIH ImageJ (https://imagej.nih.gov/ij/)(http://www.rsbweb.nihgov/ij/).

### Soybean hairy root induction and nodulation assay with chimerical plants

To generate soybean hairy roots, *A. rhizogenes* strains K599 harboring pB2GW7-*GmMAX3b* for overexpressing*,* pB7GWIWGII-*GmMAX3b* for knockdown or *GUS* were grown on LB-agar medium at 28 °C, with spectinomycin and streptomycin as selection for transformation of soybean cultivar “Tianlong No.1”, according to methods as described previously [34]. Briefly, the young soybean seedlings were wounded at hypocotyls and incubated with *A. rhizogenes* for 24 h. The infected seedlings were grown in autoclaved soils at 25 °C for 1 week till hairy roots appeared on the wounding sites. About 1 week after hairy root emergence, the chimerical soybean plants were inoculated with rhizobia strain *Bradyrhizobium japonicum* strain USDA110, which were grown in the YMA on 28 °C and the OD600nm of rhizobia was adjusted at 0.8–1.0. The rhizobia bacteria were applied about 25 ml to each plant. After 4 weeks of rhizobium application, the hairy roots and nodules were examined and collected for RNA and hormone analyses. For each binary vector including *GUS* control, at least three independent in vitro transformation experiments with the identical treatments and growth conditions were carried out.

### RNA isolation, cDNA library construction for Illumina deep sequencing

Total RNA was extracted with Trizol reagent (Invitrogen, CA, USA) or RNA kit (Biotech, Beijing) following the manufacturer’s instructions. RNA integrity was confirmed by using the 2100 Bioanalyzer. A total of 0.5–2 μg RNA per sample was used for cDNA library preparation using the TruSeq RNA sample preparation kit (Illumina, CA, USA). Each library was sequenced on an Illumina HiSeq2500 instrument and data analyses were carried out by the Biotech Company Novogene Corporation, as previously described. Approximately 70 million 100 bp pair-end reads were generated for each sample. The fragments per kilobase of transcript per million mapped reads (FPKM) and transcript level per million count values were calculated using eXpress. Differential gene expression was analyzed by using the DESeq (2012) R package. Hierarchical cluster analysis based on the differentially expressed genes (DEGs) were filtered with expression levels FPKM >5, false discovery rate < 0.01, log2 fold change >1 or <−1 in each pairwise comparison.

### Analysis of gene expression

Quantitative and semi-quantitative reverse transcriptase-PCR (qRT-PCR) analysis of gene expression was conducted as described previously [34]. Total RNAs from various tissues (Seed, root, nodule, stem, leaf and flower) of soybean seedlings or leaf of Arabidopsis plants were isolated using TRIzol reagent (Invitrogen, Carlsbad, CA) or RNA isolation kit (Biotech, Beijing) according to the manufacturer’s instructions. For each sample, 10 μg of total RNA were digested with RNase-free DNaseI (Promega, Madison, WI, USA) to remove any genomic DNA contamination. After DNaseI treatment, RNA concentration was determined again using a NanoDrop ND-2000 UV spectrophotometer (Thermo Scientific, USA). First-strand cDNA was synthesized from 2 μg total mRNA using the Superscript III first strand synthesis system (Invitrogen, CA, USA). For semi-quantitative RT-PCR, the specific primers spanning the full-length ORF of *GmMAX* genes were used. The amplification of *AtACTIN8* (*AtACT8*) was used as internal control. For the examination of expression of *GmMAX* genes in Arabidopsis transgenic lines, *GmMAX* gene-specific primers were used for quantitative RT-PCR are listed in Additional file 1: Table S1. qRT-PCR reactions were performed in 96-well plates (iQ5 Real Time PCR System; Bio-Rad) for all tissues tested, and data were analyzed.

### Hormone quantification analysis

Extraction of hormones from roots and shoots for LC-MS analysis were done as previously described [64]. In brief, 0.5 g of root or shoot samples was ground in a mortar with liquid nitrogen. The samples were extracted with 2 ml of cold ethyl acetate in a 10-ml glass vial. The vials were vortexed and sonicated for 20 min in cold water bath. Samples were centrifuged for 10 min at 2500 g at 4 °C after which the organic phase was carefully transferred to a 4-ml glass vial. The pellets were re-extracted with another 2 ml of ethyl acetate. The combined ethyl acetate fractions were dried under a flow of nitrogen gas and the residue dissolved in 250 μl of acetonitrile: water: formic acid (25: 75: 0.1, v: v: v). Before analysis, samples were filtered through Minisart SRP4 0.45 lm filters (Sartorius, Goettingen, Germany) and LC-MS ⁄MS was performed as described.

### Subcellular localization of GmMAX3b

To monitor the transient expressions of fusion proteins, the constructs in *A. tumefaciens* strain EHA105 were transformed into *N. benthamiana* leaf epidermal cells by infiltration [34]. The ORF of *GmMAX3b* in pDONR221 was recombined into pK7WGF2 in fusion with GFP at N-terminal by using Gateway recombination LR Clonase (INVITROGEN). The sequencing-confirmed GFP-GmMAX3b vectors were transformed into *A. tumefaciens* strain GV3101 through electroporation. The positive colony was grown at 28 °C overnight and re-suspended in infiltration medium (10 mM MES, pH 5.6, 10 mM MgSO_4_, and 100 mM acetosyringone) for transformation of tobacco. The infiltrated tobacco leaves were checked for GFP signals of fusion proteins after 2 days of incubation. The Olympus BX53 microscope and Leica Sports cofocal microscope were used to imaging at an excitation wavelength of 488 nm and emissions collected at 500–530 nm filter to record GFP images and 650–700 nm to record chloroplast autofluorescence [34].

### Bioinformatics analysis

GmMAX protein sequences obtained from sequencing of our clones were deposited onto GenBank with accession numbers: *Glycine max* GmD27a (KY486796), GmD14a (KY486797), GmMAX1a (KY486798), GmMAX1b (KY486799), GmMAX2a (KY486800), GmMAX3b (KY486801), GmMAX4a (KY486802), and GmMAX4b (KY486803). Amino acid multiple alignments were made with the ClustalW program (http://www.ebi.ac.uk/clustalw/) under default parameters. A phylogenetic tree was constructed using MEGA6. The significance level of the neighbor-joining analysis was examined by bootstrap testing with 1000 repeats.

### Statistical analysis

Most data was recorded from at least three independent experiments and Student’s *t*-test was applied to analyze the difference in data comparisons. The confidence limit 95% represents the significant between two tailed data.

## Results

### Identification of AtMAX homologues from soybean genome

Arabidopsis AtMAX1, 2, 3, 4 or rice DWARF /HTD homologue protein sequences were used for blast against soybean genome (https://phytozome.jgi.doe.gov/pz/portal.html). This research resulted in the identification of at least two close homologues for each of AtMAX1, 2, 3, and 4. GmMAX1a and GmMAX1b were encoded by Glyma.04 g05510 and Glyma.06 g05520.2 loci, respectively (Additional file 2: Figure S1). Phytozome database show that *GmMAX1a* has three transcript variants and *GmMAX1b* has four transcript variants. Both GmMAX1a and GmMAX1b showed approximately 84–5% similarity and 69% identity with AtMAX1 at protein sequence. GmMAX1a and GmMAX1b proteins shared approximately 90% identity with each other. The two soybean homologues of AtMAX2 are encoded by loci Glyma12g15360.1 and Glyma06g43000.1, designated as GmMAX2a and GmMAX2b respectively (Additional file 3: Figure S2). GmMAX2a showed approximately 71% similarity and 59% identity with AtMAX2 at the amino acid level. GmMAX2a shows approximately 99% similarity and 59% identity with AtMAX2 protein. GmMAX2a and GmMAX2b shared 97% similarity and 92% identity with each other and *GmMAX2a* is expressed higher than these of *GmMAX2b*. Arabidopsis MAX3 (AtMAX3) has also two closest homologues in soybean genome encoded by loci Glyma01g14266.1 and Glyma11g16370.1, designated as GmMAX3a and GmMAX3b, respectively (Additional file 4: Figure S3). GmMAX3b showed approximately 77% similarity and 62% identity with AtMAX3 at the amino acid level. *GmMAX3a* is expressed at much lower level than GmMAX3b does; the expression patterns of *GmMAX3b* are also most similar to these of *AtMAX3*, we therefore only studied GmMAX3b. Arabidopsis MAX4 (AtMAX4) has two homologues in soybean genome encoded by loci Glyma04g08910.1 and Glyma06g09000.2, designated as *GmMAX4a* and GmMAX4b, respectively (Additional file 5: Figure S4). The primary variant of GmMAX4b showed approximately 78% similarities and 65% identity with AtMAX4. GmMAX4a and GmMAX4b shared approximately 97% similarity and 93% identity with each other at protein sequence level.

### Tissue specific expression patterns of *GmMAX* genes

**Fig. 1:**
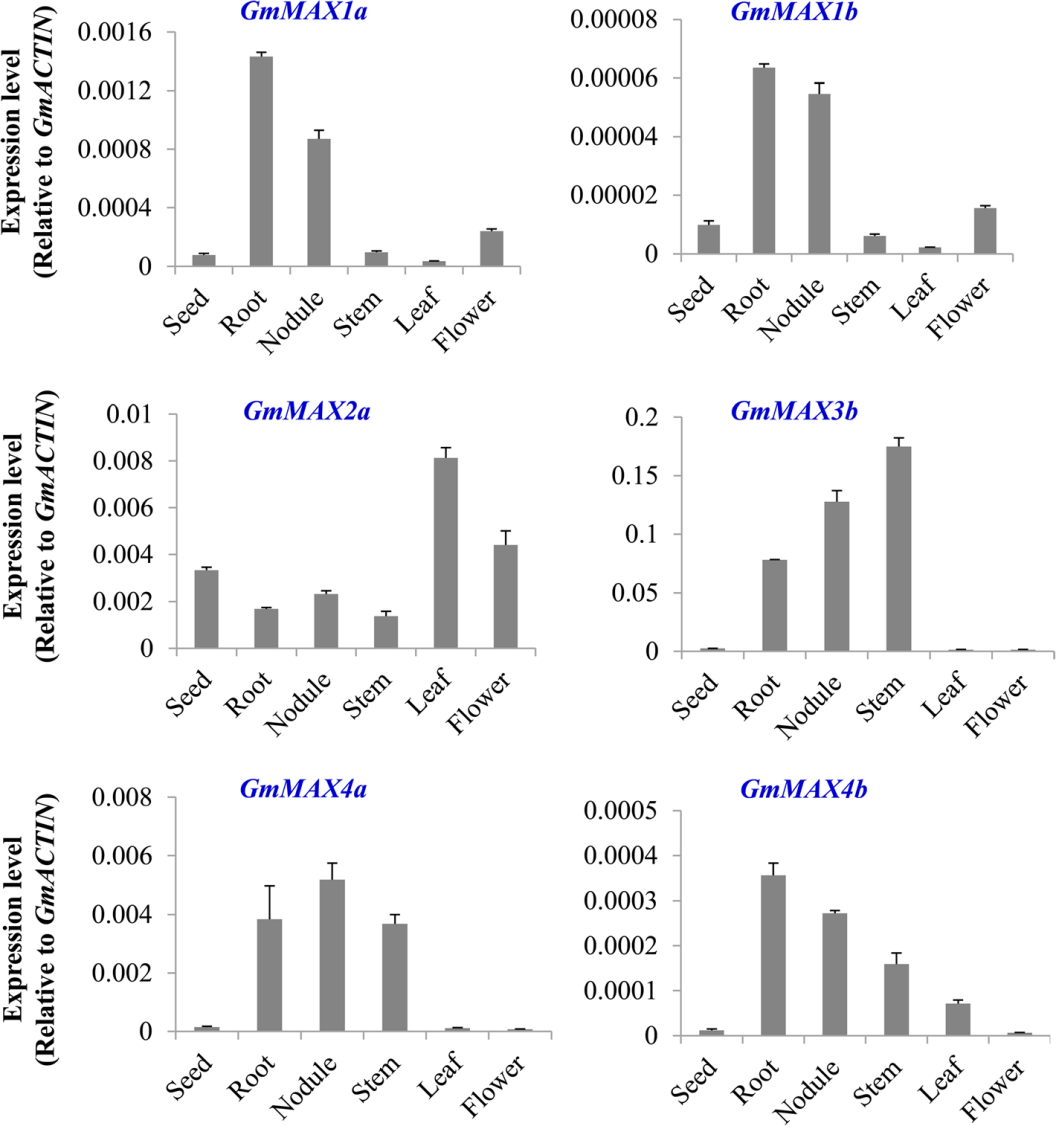
Tissue-specific expression patterns of *GmMAX1a, 1b, 2a, 3b,* and *4a*. Relative expression of these *GmMAXs* to *GmACTIN* was measured with samples from soybean cultivar “Tianlong 1” at different development stages by using qRT-PCR. Data are expressed as means ± s.d from 3 independent experiments with biological replicates

We examined the expression patterns of each *GmMAX* gene across various tissues and organs in soybean plants. *GmMAX1a* transcripts were at the highest level in root and nodules and the expression of *GmMAX1a* in the rest of other tissues was low (Fig. 1 **)**. *GmMAX1b* was also highly expressed in nodule and root, where *GmMAX1a* transcripts were more than 6 times greater than in stem and seeds (Fig. 1). *GmMAX1a* was expressed over 20-fold higher than *GmMAX1b* across most tissues and organs (Additional file 6: Figure S5). *GmMAX2a* were also expressed in most tissues, but with the highest expression levels in leaf and flower and seed. *GmMAX3b* was highly and more specifically  expressed in soybean stem, nodule, and stem.Both GmMAX4a and GmMAX4b displayed highest expression levels in root, nodule, and stem. But GMMAX4a transcripts levels were much higher than these of GmMAX4b, suggesting that GmMAX4a should be the primary copy in the function (Fig. 1 **).**

*GmMAX4a* was expressed to an extremely high level in the stems, and then in root and nodules. *GmMAX4b* was highly expressed in nodules, then in stem and root. Both GmMAX4a and b had low expression levels in the rest of other tissues **(**Fig. 1). GmMAX4a was expressed over 10-fold higher than *GmMAX4b* across most tissues and organs, which is consistent with transcriptomic data from public database (Additional file 6: Figure S5).

### Functions of GmMAX1a when expressed in Arabidopsis

In order to test whether *GmMAX* genes function similarly as corresponding *AtMAX* ortholog genes, we conducted genetic complementation by expressing *GmMAX* genes, driven by the constitutive 35S promoter, in corresponding Arabidopsis max mutants. We focused on a number of primary phenotypes that were observed with these mutants, to determine whether heterogonous expression of soybean *GmMAX* gene could rescue the corresponding Arabidopsis *max* mutants.

**Fig. 2:**
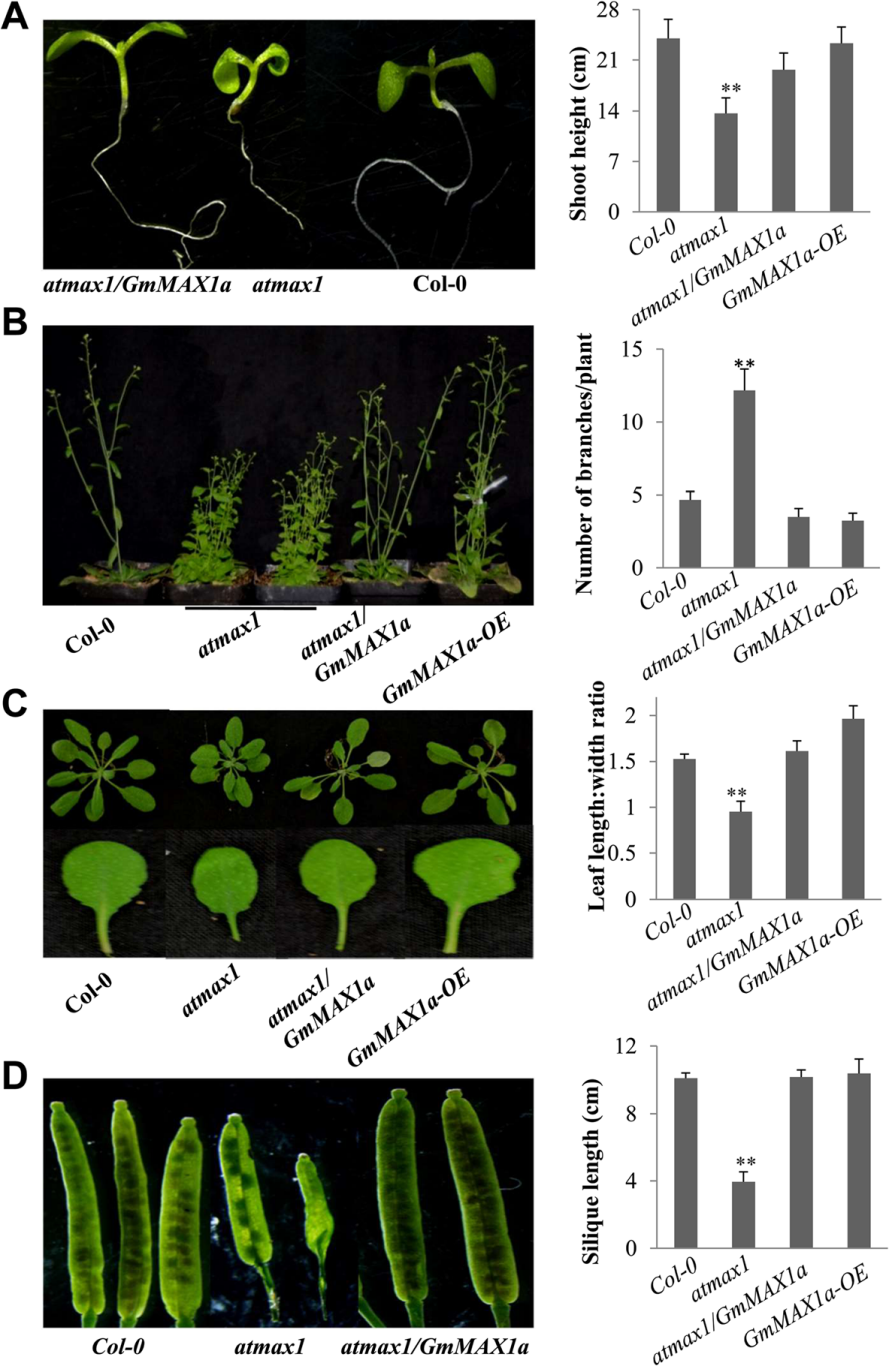
Function analysis of *GmMAX1a* expressed in Arabidopsis. Complementation of *GmMAX1a* in *atmax1–1* mutant and overexpression in the wild-type Col-0 were done to compare the functions of GmMAX1a with AtMAX1. **a** The seedlings of *atmax1–1,* Col-0, and *atmax1–1* complementation plant with *GmMAX1a* (*atmax1/GmMAX1a*) with different primary root lengths (left panel). Quantification of shoot height was conducted and calculated on at least three independent lines of more than 20 plants (right panel). **b** Mature plants of Col-0, *atmax1–1* mutant, complementation (*atmax1/GmMAX1a*), and *GmMAX1a* overexpression lines (left panel). The numbers of branches were determined and calculated on at least three independent lines of more than 20 plants (right panel). **c** The differences between seedlings (top panel) and leaf shapes (bottom panel) of Col-0, *atmax1–1,* complementation of (*atmax1–1/GmMAX1a*) and *GmMAX1a-OE* plants (left panel). Ratios of leaf length to leaf width were collected on 2-week-old seedlings (right panel). Data represent means ± s.d (*n* = 20 plants for each genotyping). **d** Silique lengths of Col-0, *atmax1–1,* and complementation of (*atmax1/GmMAX1a*) plants (left panel). Quantification of silique length (cm) of Col-0, *atmax1–1,* complementation of (*atmax1/GmMAX1a*) and *GmMAX1a-OE* plants (right panel). Data represent means ± s.d (*n* = 20 plants for each genotyping). Data are expressed as means ± s.d. from 3 independent experiments with biological replicates. Asterisks indicate significant difference according to a Student’s *t*-test (**P < 0.001, * *P* < 0.05)

AtMAX1 is essentially involved in SL biosynthesis and the loss-of-function of AtMAX1 caused smaller leaf blade size, shorter primary root, reduced height and more branches, than wild-type Col-0 (Fig. 2a, b) [11], whereas 35S:*:GmMAX1a* transgenic *atmax1* plants restored the leaf blade size to Col-0. We verified all transgenic Arabidopsis lines; the Col-0 and *atmax1* mutants showed no GmMAX1a transcripts while the complement 35S::*GmMAX1a* transgenic lines showed strong expression (Additional file 7: Figure S6A). GmMAX1a could rescue *atmax1* defective phenotype in leaf shape development; leaves of *atmax1* mutant were rounder than those of Col-0, whereas 35S::*GmMAX1a* plants showed very similar leaf shape with Col-0 plants (Fig. 2c). The most typical phenotype of *atmax1* mutant plants is more auxiliary branches as compared with Col-0,a significant reduction in the branching number, about 3 to 7 in average, was observed when *GmMAX1a* was introduced into *atmax1* mutant or overexpression of *GmMAX1a* in the Col-0 background (Fig. 2b). Under normal growth conditions, 45-day old *atmax1* mutants were shorter than the wild-type Col-0, and GmMAX1a restored the mutant’s shoot height to the wild-type level (Fig. 2b). The siliques of *atmax1* mutant plants were shorter with less seed than these of Col-0, whereas overexpression of *GmMAX1a* in *atmax1* completely recovered this phenotypes (Fig. 2d). Therefore, *GmMAX1a* expression completely rescued the more auxiliary branches and lower shoot height phenotypes that are solely due to loss-of-function of *AtMAX1*. These results demonstrated that *GmMAX1a* and *AtMAX1* possess conserved function in plant development and growth.

### Functional analysis of *GmMAX2a* in Arabidopsis

**Fig. 3:**
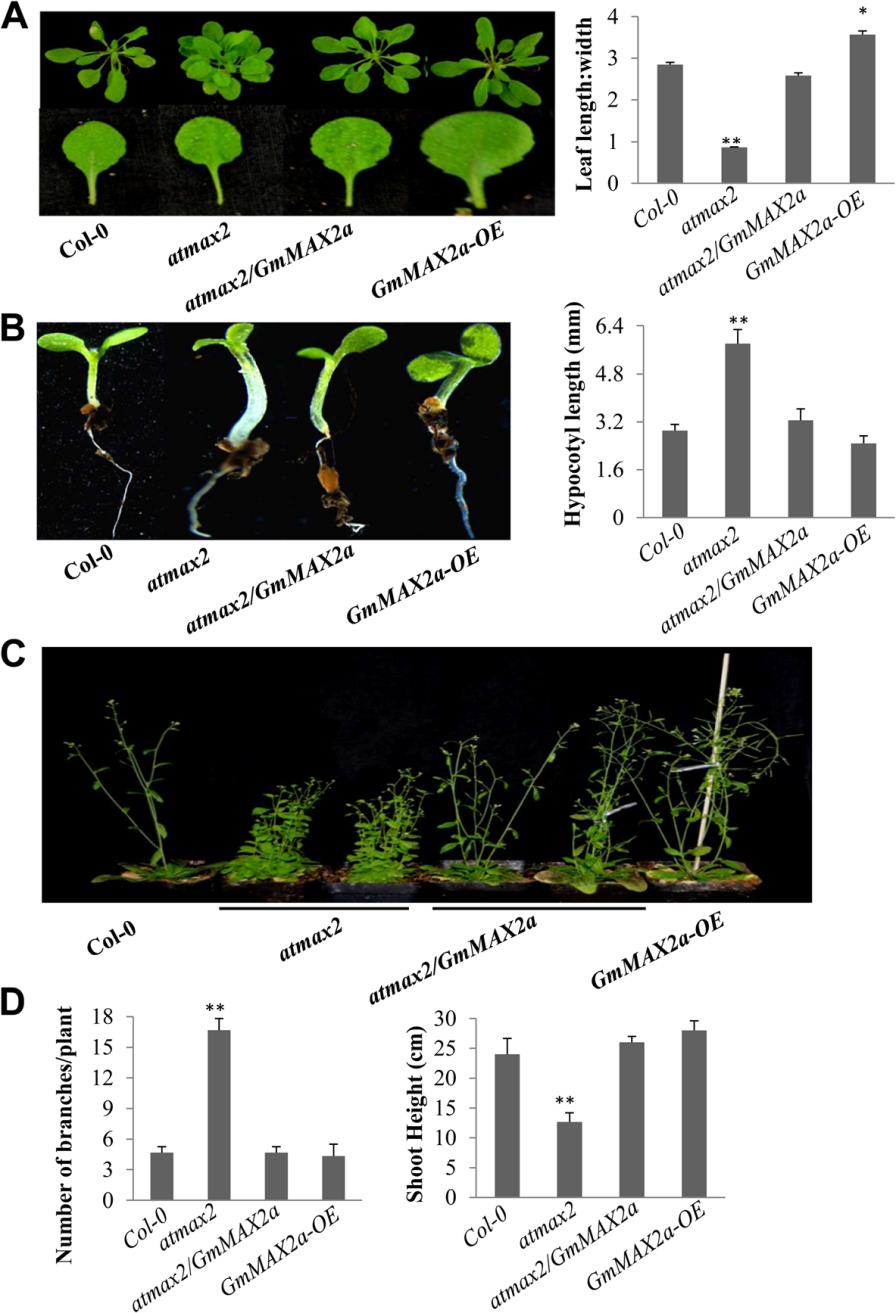
Functions of *GmMAX2a* expressed in Arabidopsis plants. **a** The differences between seedlings (top panel) and leaf shapes (bottom panel) of Col-0, *atmax2,* complementation of (*atmax2/GmMAX2a*) and *GmMAX2a-OE* plants (left panel). Ratios of leaf length to leaf width were collected on 2-week-old seedlings (right panel). Data represent means ± s.d (*n* = 20 plants for each genotyping). **b** Hypocotyl lengths of wild type (Col-0), *atmax2,* mutant complementation (*atmax2/GmMAX2a*) and *GmMAX2a-OE* plants. Data represent means ± s.d (*n* = 30 seedlings for each genotyping). **c** Mature plants of Col-0, *atmax2*, mutant complementation (*atmax2/GmMAX2a*), and *GmMAX2a* overexpression lines. **d** Quantification of the number of branches/plant (left panel) and shoot height (cm) (right panel) was conducted and calculated on at least three independent lines of more than 20 plants. Data are expressed as means ± s.d. from 3 independent experiments with biological replicates. Asterisks indicate significant difference according to a Student’s *t*-test (**P < 0.001, * *P* < 0.05)

F-box protein AtMAX2/OsD3/PtRMS4 is essentially involved in SL signal perception and transduction. Arabidopsis *atmax2* mutant displays an auxiliary branch phenotype as other *atmax* mutants [54], reduce root hairs, delayed seed germination [43, 50], and an increased hypocotyls length in light-grown seedlings [59]. We confirmed these phenotypes of *atmax2* in our growth conditions as compared with the wild-type control (Fig. 3). We generated *GmMAX2a* transgenic lines in Col-0 and *atmax2* mutant backgrounds to texted its function. The Col-0 and *atmax2* mutants showed no *GmMAX2a* transcript while the 35S::*GmMAX2a* transgenic lines showed much more *GmMAX2a* transcripts (Additional file 7: Figure S6B). Analysis of most independent homozygous T3 transgenic lines showed that Gm*MAX2a* overexpression can inhibit the auxiliary branching numbers of *atmax2* mutant (Fig. 3c). Under the identical conditions, wild-type Col-0 plants had an average of 5 branches, while the *atmax2* mutant had ~16 braches. The *atmax2* mutant expressing *GmMAX2a* had clearly reduced branch number, similar to that of the wild-type (Fig. 3d). While the *35S::GmMAX2a* overexpression in wild-type caused no difference in branch numbers with the wild-type Col-0 (Fig. 3d). The *GmMAX2a* overexpression plants have shoot heights similar to these of wild-type Col-0, and were significantly higher than the *atmax2* mutant plants (Fig. 3c, d). The hypocotyls of *35S*::*GmMAX2* transgenic *atmax2* plants became shorter than those in *atmax2* and approximately resembled to Col-0, suggesting that the hypocotyls of *atmax2* mutants were restored by GmMAX2a (*P* < 0.01) (Fig. 3b). The hypocotyl lengths of *GmMAX2a* overexpression in wild-type were also significantly shorter (*P* < 0.01) than the wild-type. The leaf shape phenotypes of *atmax2* mutant were also rescued by overexpression of *GmMAX2a* in the *atmax2* mutant background (Fig. 3a)**.**

### Functional analysis of *GmMAX3b* in Arabidopsis

**Fig. 4:**
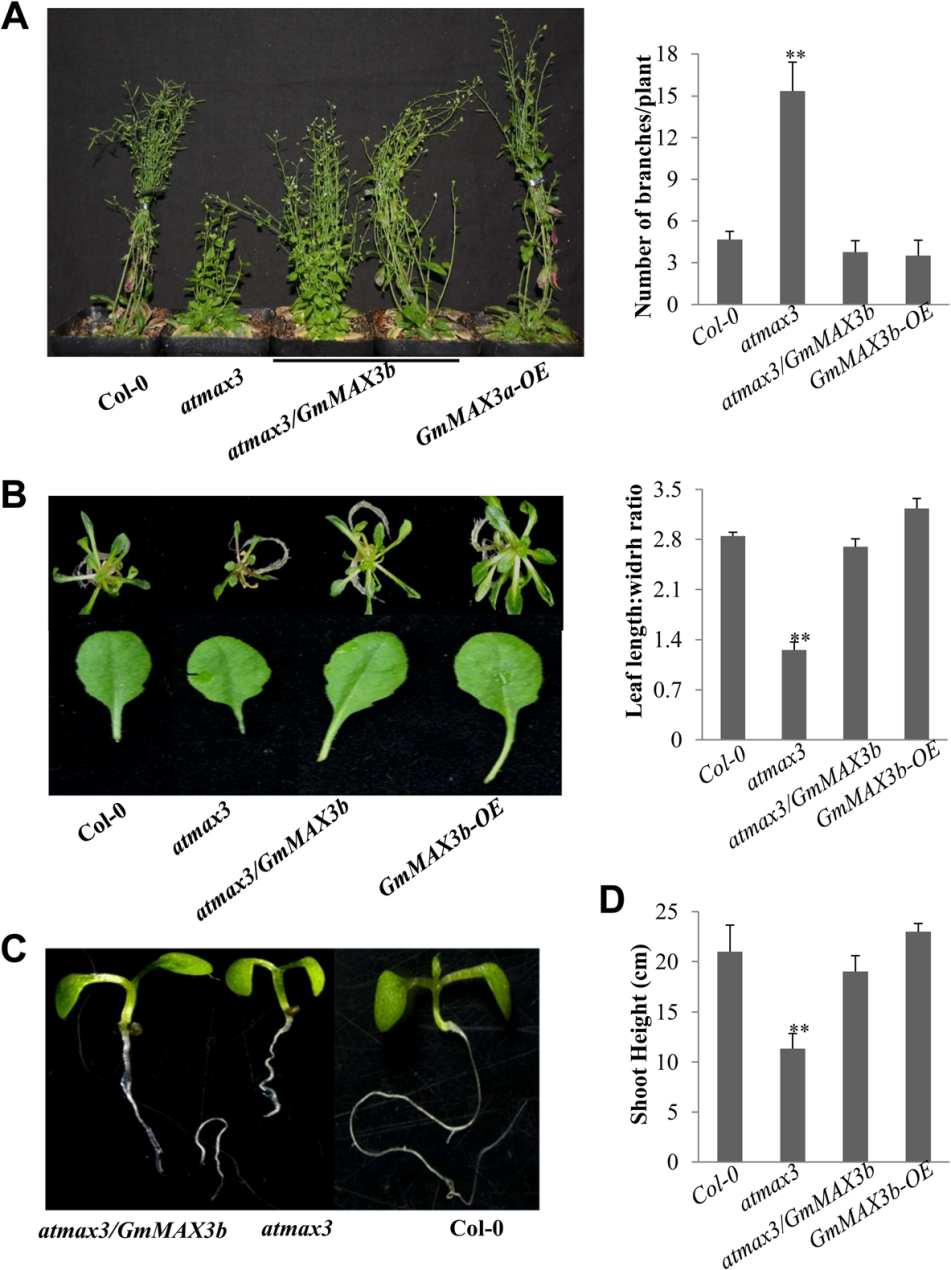
Functions of GmMAX3b when expressed in Arabidopsis plants. **a** Mature plants of Col-0, *atmax3–9,* mutant complementation (*atmax3/GmMAX3b*), and *GmMAX3b* overexpression lines. Plants were photographed at 35 days after sowing (left panel). Quantification of the number of branches of each plant (right panel) was conducted and calculated on at least three independent lines of more than 20 plants. **b** The differences between seedlings (top panel) and leaf shapes (bottom panel) of *Col-0*, *atmax3,* mutant complementation plant (*atmax3/GmMAX3b*), and *GmMAX3b-OE* plants (left panel). Ratios of leaf length to leaf width (right panel) were collected on 2-week-old seedlings. Data represent means ± s.d (*n* = 20 plants for each genotyping). **c** The different primary root lengths seedlings of *atmax3/GmMAX3b* complementation*, atmax3,* and Col-0 plant with *GmMAX3b* (*atmax3/GmMAX3b*). **d** Quantification of shoot height was conducted and calculated on at least three independent lines of more than 20 plants. Data represent means ± s.d from three independent experiments. Asterisks indicate significant difference according to a Student’s *t*-test (**P < 0.001, * *P* < 0.05)

GmMAX3, a putative carotenoid cleavage dioxygenase 7, is ortholog to Arabidopsis AtMAX3, rice HTD1, petunia DAD3 or pea RMS5 involved in SL biosynthesis [10, 76]. *GmMAX3b* was ectopically expressed in *atmax3–9* mutant and wild-type Col-0 backgrounds for examination of its function. The Col-0 and *atmax3–9* mutants showed no *GmMAX3b* transcript while the 35S::*GmMAX3b* complement transgenic lines showed stronger expression (Additional file 7: Figure S6C). Compared with *atmax3–9* mutant plants, the number of primary rosette-leaf branches in *GmMAX3b/atmax3* transgenic lines was significantly reduced, suggesting that *GmMAX3b* was able to complement the shoot branching phenotype of *atmax3–9* mutant plants. While the *GmMAX3b* overexpression lines of Arabidopsis did not show any difference in the rosette branch number from the wild-type (Fig. 4a)**.** *GmMAX3b* also complemented the reduced shoot height of *atmax3–9* as compared to the wild-type, as well as the short primary roots of *atmax3–9* seedlings to those of the wild-type (Fig. 4c).The overexpression of *GmMAX3b* slightly increased the shoot height as compared to the wild-type, but restored the long primary roots of *GmMAX3b/atmax3* seedlings (Fig. 4c). The leaf shape phenotypes of *atmax3–9* mutant were also rescued by overexpression of *GmMAX3b* in the mutant background (Fig. 4b, d).

### Genetic complementation of Arabidopsis *atmax4–1* by *GmMAX4a*

**Fig. 5:**
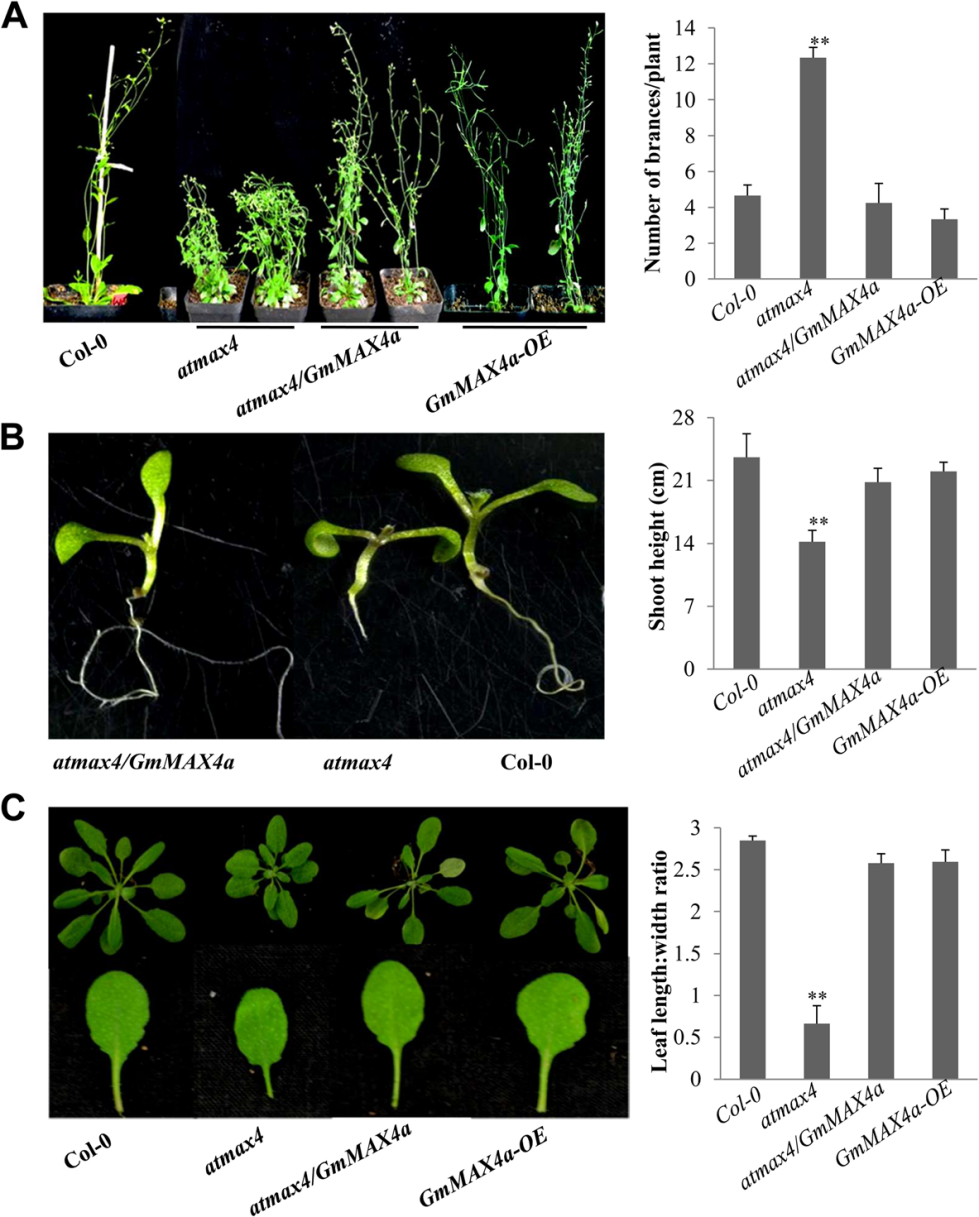
Functions of GmMAX4a when expressed in Arabidopsis. **a** Mature plants of Col-0, *atmax4* mutant, complementation (*atmax4/GmMAX4a*), and *GmMAX4a-OE* lines. Plants were photographed at 25 days after sowing (left panel). Quantification of the number of branches of each plant (right panel) was conducted and calculated on at least three independent lines of more than 20 plants. **b** The different primary root lengths seedlings (left panel) of *atmax4* complementation plant with *GmMAX4a* (*atmax4/GmMAX4a*), *atmax4* and Col-0 plants. Quantification of the shoot height (right panel) were conducted and calculated on at least three independent lines of more than 20 plants. **c** Leaf shapes of Col-0, *atmax4,* complementation (*atmax4/GmMAX4a*), and *GmMAX4a-OE* plants (left panel). Ratios of leaf length to leaf width were collected on 2-week-old seedlings (right panel). Data represent means ± s.d (*n* = 20 plants for each genotyping). Data represent means ± s.d from three independent experiments. Asterisks indicate significant difference according to a Student’s *t*-test (**P < 0.001, * *P* < 0.05)

AtMAX4, ortholog to rice DWARF10, pea RMS1, or petunia DAD1, encoding a carotenoid cleavage dioxygenase 8 (CCD8) involved in SL biosynthesis [51, 52]. The *atmax4–1* mutant showed more auxiliary branches and reduced shoot height, as compared to the wild-type Col-0, due to the deficiency of SL biosynthesis [51, 52]. We generated *35S:GmMAX4a* transgenic lines in both *atmax4* mutant and wild-type backgrounds (Fig. 5). The Col-0 and *atmax4* mutants showed no *GmMAX4a* transcript while the complement 35S::*GmMAX4a* transgenic lines showed more *GmMAX4a* transcripts (Additional file 7: Figure S6D). Compared with *atmax4* mutants, the numbers of primary rosette-leaf branches were significantly reduced in these *GmMAX4a-*complementation lines, and almost completely restored to the wild-type’s branch numbers (Fig. 5a). However, *GmMAX4a* overexpression lines had not obviously further inhibited the branch number of the wild-type. *GmMAX4a-*complementation plants also appeared to be taller than *atmax4* mutant plants, but the overexpression lines of *GmMAX4a* were not significantly higher than the wild type (Fig. 5a). The primary root lengths of *GmMAX4a-*complementation seedlings were much longer than that of *atmax4* mutant, and similar to the wild-type seedlings (Fig. 5b). In terms of leaf development and leaf shape, *GmMAX4a-*overexpression also restored the short petiole and round shape of *atmax4* mutant to the wild-type (Fig. 5c).

### *GmMAX3b* complemented the *atmax3* mutants’ defective root hair phenotype

**Fig. 6:**
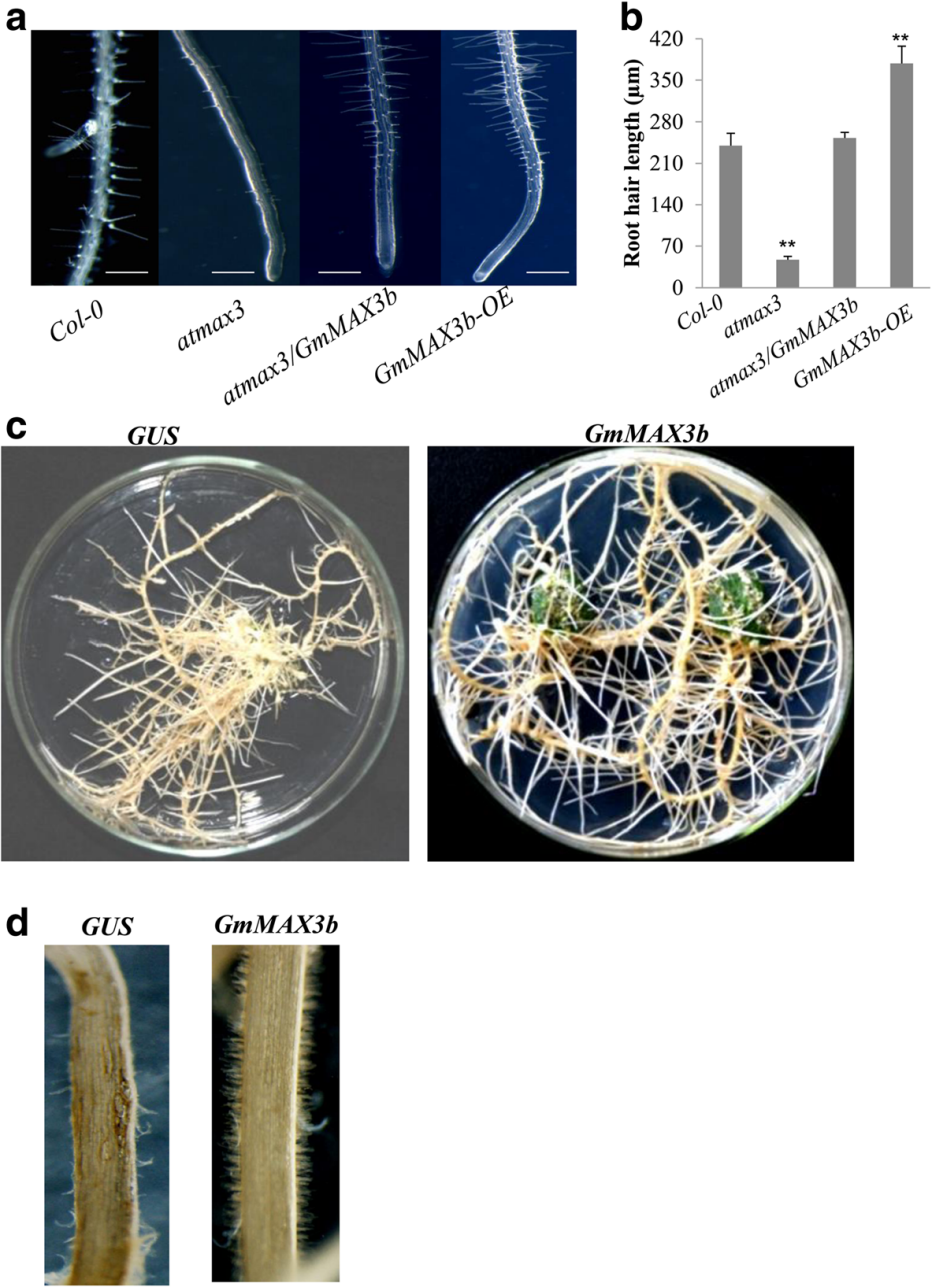
Effects of *GmMAX3b-*overexpression on morphology of hairy roots in mutant Arabidopsis and Soybean. **a** Root hairs of Col-0, *atmax3–9*, and complementation (*atmax3/GmMAX3b*) plants. Bar = 1 mm. **b** Quantification of root hairy length of Col-0, *atmax3–9*, and complementation (*atmax3/GmMAX3b*) plants. **c** Soybean hairy roots expressing GmMAX3b and *GUS* (control). **d** Root hair patterns in the soybean hairy roots overexpressing *GmMAX3b* or *GUS*. Data represent means ± s.d (*n* = 20 roots for each genotyping). 20 root hairs of each root were used for determination of root hair length

As for the root growth, the knockout mutation in *max3* resulted in shorter primary root and root hair lengths, but longer lateral root length [28, 30]. We also tested root phenotypes of the overexpression lines for *GmMAX3b in atmax3* mutants, respectively. *35S: GmMAX3b* transgenic plants showed the increased primary root and root hair length (Fig. 6a, b)**,** suggesting that *GmMAX3b* can complement the primary root and root hair defects in these mutants.

The *GmMAX3b*-*OE* also increases the root hairs in the soybean transgenic hairy roots in vitro (Fig. 6c). The observation of transgenic hairy roots under microscope suggested that *GmMAX3b-OE* had developed more and better root hairs than the *GUS* control, as suggested by the higher root hair density and longer root hairs than the *GUS* control (Fig. 6d).

### *GmMAX* expression in Arabidopsis affected hormone levels

**Fig. 7:**
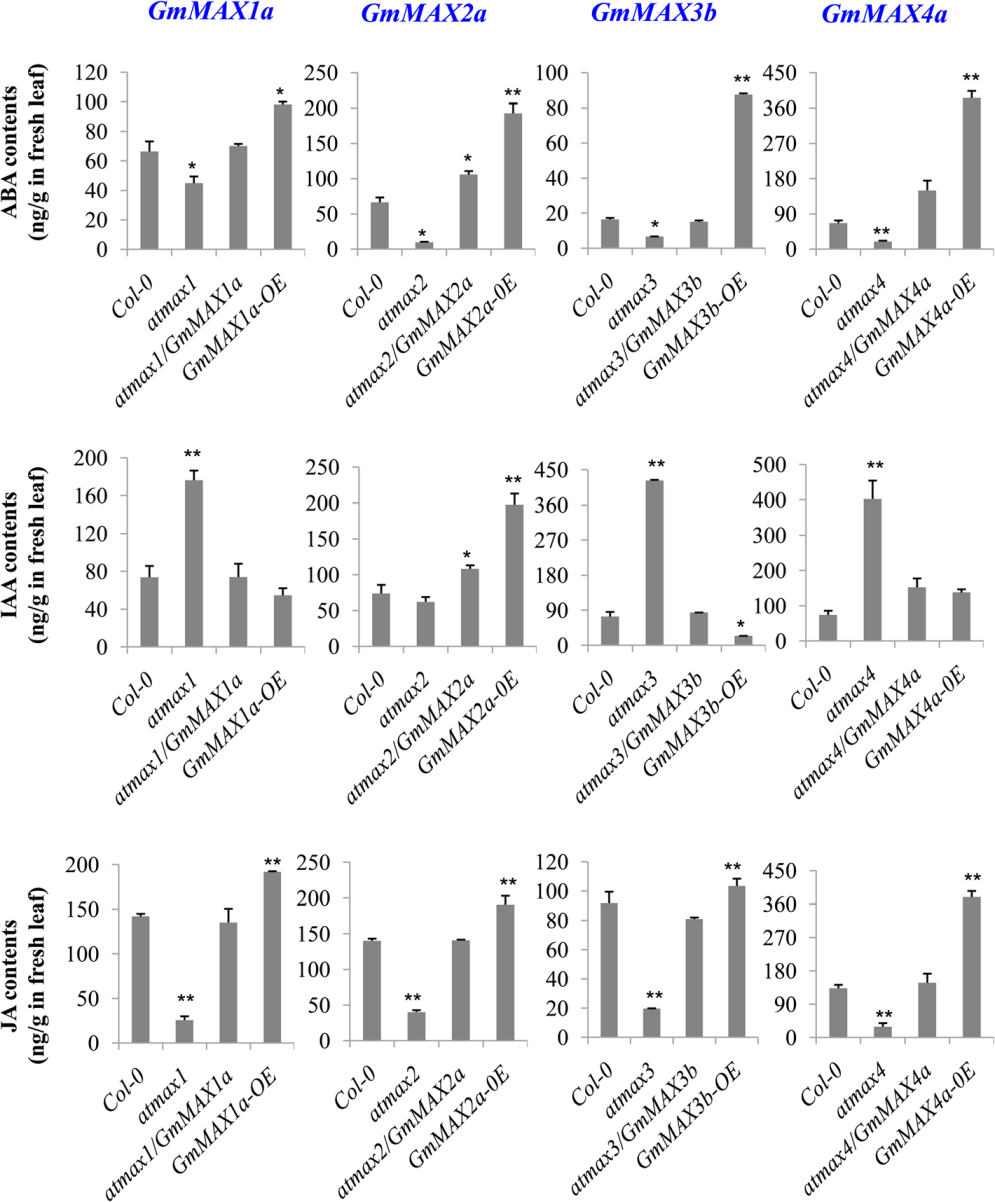
Hormone analyses on *GmMAX*s-transgenic Arabidopsis plants. Quantification of hormones IAA, ABA, JA (both free plus JA-Ile) in leaves of wild-type Col-0, *atmax1, atmax2 atmax3,* and *atmax4* mutants, and their complementation (*atmax1/GmMAX1a*, *atmax2/GmMAX2a*, *atmax3/GmMAX3b*, and *atmax4/GmMAX4a*), and overexpression (*GmMAX1a-OE, GmMAX2a-OE, GmMAX3b-OE,* and *GmMAX4a-OE*) plants, respectively. Data are expressed as means ± s.d (*n* > 2). Asterisks indicate significant difference between wild-type control, max mutants, complementation, and overexpression lines, according to a Student’s *t*-test (**P < 0.001, * *P* < 0.05)

One important role of *MAX* genes as SL biosynthesis and signaling components is that they affect various other hormones, through which SLs exerted their physiological functions on different physiological processes [31, 49]. We measured hormones in the fresh leaves of Arabidopsis plant in various genetic backgrounds. It was found that the *atmax1, atmax2, atmax3*, and *atmax4* mutant plants had significantly reduced ABA and JA contents as compared with the wild-type (Fig. 7). Moreover, except for *atmax2,* other mutants such as *atmax1, atmax3*, and *atmax4* had significantly increased IAA levels as compared with the wild-type (Fig. 7). While each complementation line had significantly rescued ABA and JA contents as, compared with the *atmax1, atmax2, atmax3*, and at*max4* mutant plants and wild-type Col-0 plants. The IAA levels in *atmax1, atmax3*, and *atmax4* complementation plants, *atmax1/GmMAX1a, atmax3/GmMAX3b*, and *atmax4/GmMAX4a* plants, respectively, had also reduced almost to the wild-type levels (Fig. 7). Meanwhile, the overexpression lines of *GmMAX1a, GmMAX2a, GmMAX3b* and *GmMAX4a* had increased ABA and JA contents, but reduced IAA levels, as compared to the wild type**.** The overexpression of *GmMAX1a, GmMAX2a, GmMAX3b, and GmMAX4a* in their corresponding orthologous mutants and the wild-type Col-0 had promoted the biosynthesis of ABA and JA (Fig. 7). These data demonstrated that overexpression of *GmMAXs* affected the hormone levels in these transgenic Arabidopsis plants, and this may be the major mechanisms, by which these GmMAXs rescued *atmax* mutant phenotypes. With an exception, *atmax2* and its complementation line *atmax2/GmMAX2a*, and *GmMAX2a* overexpression lines did not shown significantly increased IAA contents as compared with the control wild-type (Fig. 7).

### Overexpression and knockdown of *GmMAX3b* in soybean hairy roots altered nodulation

**Fig. 8:**
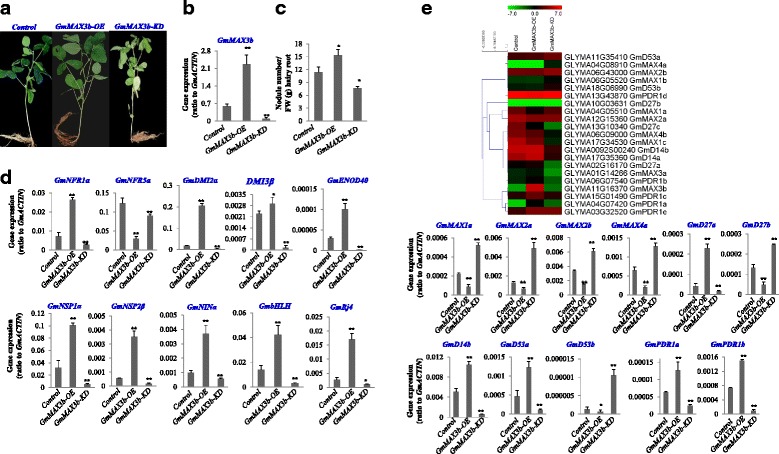
Effect of *GmMAX3b* overexpression and *GmMAX3b* knockdown in hairy roots on soybean nodulation. Chimerical soybean plants were generated by transformation with K599 harboring, *GmMAX3b*-overexpression (*GmMAX3b-OE)*, *GmMAX3b*-knockdown (*GmMAX3b-KD)* or *GUS* vector. Plants with wild-type shoots and transgenic hairy roots were inoculated with *Bradyrhizobium japonicum* strain USDA110. Nodule numbers from the hairy roots were counted and the roots were sampled for gene expression analysis at 28 days post inoculation. **a** Chimerical soybean plants with wild-type shoots but transgenic hairy roots. *GmMAX3b-OE* plants developed more nodules as compared with *GUS* control. **b** qRT-PCR confirmation of *GmMAX3b*-*OE* and *GmMAX3b-KD* in transgenic hairy roots as compared to GUS control. **c** Hairy root fresh weight (g) and nodule numbers ratio in *GmMAX3b-OE, GmMAX3b-KD* and *GUS* control lines. **d** Expression levels of nodulation genes in *GmMAX3b-OE, GmMAX3b-KD* and *GUS* transgenic hairy roots as control. **e** Validation of SL biosynthesis and signaling genes in *GmMAX3b-OE, GmMAX3b-KD* and *GUS* as control hairy root lines. Gene expression was determined by qRT-PCR with *GmACTIN* as an internal control. Data are expressed as means ± s.d. from at least 3 independent experiments with biological replicates. Differences were analyzed, *p < 0.05; **p < 0.01 in student’s *t*-test

We then examined the functions of GmMAX3b in soybean nodulation. By transformation of wild-type soybean hypocotyls to generate hairy roots, we generated chimerical transgenic soybean plants with wild-type shoots and transgenic hairy roots overexpression and knockdown of *GmMAX3b* ***.*** In comparison with similar chimerical soybean transgenic plant with hairy roots overexpressing *GUS* as a control, *GmMAX3b-OE* plants gave more nodules (>30%) while *GmMAX3b-KD* plants developed less nodules than control (Fig. 8a, c).

In order to address how *GmMAX3b-OE* and *GmMAX3b-KD* chimerical plants produced more nodules than the *GUS* control did, we analyzed nodulation genes in these transgenic hairy roots (Fig. 8d). Some of the key genes involved in legume nodulation were expressed much higher in *GmMAX3b-OE* while expression was lower in *GmMAX3b-KD* transgenic hairy roots than in *GUS* control (Additional file 8: Data S1, Additional file 9: Figure S7), as confirmed by qRT-PCR, which also showed the up-regulation of *GmNFR1α, GmDMI2α, GmNSP2β, GmNINα, GmENOD40, GmbHLH, and GmRj4* and vice versa in *GmMAX3b*-*KD* (Fig. 8d)*.* Meanwhile, the expression of *GmNFR5α* were lower in both *GmMAX3b-OE* and *GmMAX3b*-*KD* than in *GUS* hairy root control. *GmDMI3β* showed no substantial change in *GmMAX3b-OE* but significantly decreased expression in *GmMAX3b*-*KD* and *GUS* hairy roots (*p* < 0.05, Fig. 8d). The differences on expression of these key nodulation genes may explain why *GmMAX3b-OE* and *GmMAX3b*-*KD* transgenic hairy roots had altered the nodules than the *GUS* control.

### Global gene expression changed in *GmMAX3b-OE* and *GmMAX3b*-*KD* soybean hairy roots

To learn how and what *GmMAX3b* overexpression and knockdown had changed the hairy roots in transgenic chimerical soybean plants, we did RNA-Seq in comparison to *GUS* control. The transcriptomic analysis showed that expression of more than 2000 genes was changed in *GmMAX3b-OE* hairy roots compared with *GUS* control. The expression of nodulation genes, SL biosynthesis and signaling genes was changed (Fig. 8e,Additional file 8: Data S1). Besides the significantly overexpression and knockdown of *GmMAX3b*, expression of SL biosynthesis and signaling genes, such as *GmD27a, GmD14b, GmD53a, GmPDR1a* and *GmPDR1a* was significantly up-regulated (*P* < 0.05) while *GmMAX1a, 2a, 2b,* and *4a* significantly down-regulated (*P* < 0.05) and vice versa in *GmMAX3b*-*KD* hairy roots (Fig. 8e).

Some of the key genes involved in auxin biosynthesis pathway were expressed much higher in *GmMAX3b-OE* and down-regulated in *GmMAX3b*-*KD* than in *GUS* control (Additional file 10: Figure S8). The expression of auxin biosynthesis genes, such as *YUC8a*, *YUC8b, YUC5a,* and *YUC9a* was higher while the expression levels of *YUC12a* and *YUC5b* was lower in *GmMAX3b-OE* transgenic hairy roots than in the *GUS* control, and the opposite expression patterns for these genes were observed in *GmMAX3b-KD* hairy root lines (Additional file 8: Data S1).

Complex interactions between SL signaling with other hormone signaling were revealed by transcriptome of *GmMAX3b* overexpression and knockdown hairy roots (Additional file 8: Data S1). JA biosynthetic genes, such as several allene oxide synthases *GmAOSa (*GLYMA17G36530 and *GmAOSb* (GLYMA07G21100) and 12-oxophytodienoate reductase *GmOPDR* (GLYMA01G44600), jasmonate *O*-methyltransferase *GmJAOMa* (GLYMA18G47370), as well as JA-specific GH3 genes *GmGH3s* (GLYMA01G39780), were up-regulated in *GmMAX3b-*OE and down-regulated in *GmMAX3b*-*KD* hairy roots as compared with *GUS* control. Meanwhile, more than 10 ethylene-responsive transcription factor (ERF) genes were up-regulated *in GmMAX3b-OE* and down-regulated in *GmMAX3b*-*KD* hairy roots. Carotenoid pathway genes showed differential expression patterns in *GmMAX3b-OE* and *GUS* control hairy root lines. Beta-carotene 3-hydroxylase, zeta-carotene desaturase, carotene epsilon-monooxygenase; phytoene dehydrogenase, phytoene desaturase genes did not show difference. Other genes encoding carotene epsilon-monooxygene *GmCEMa* (GLYMA13G21110), prolycopene isomerase *GmPCI* (GLYMA07G40340); xanthoxin dehydrogenase *GmXDH* (GLYMA11G21180); lycopene epsilon cyclase; violaxanthin de-epoxidase *GmVEO1* (GLYMA03G41420), *GmVEO2* (GLYMA19G44010); zeaxanthin epoxidase *GmZEO* (GLYMA17G20020) were down-regulated in *GmMAX3b-OE* and up-regulated in *GmMAX3b*-*KD* hairy roots compared to the *GUS* control. ABA catabolic genes encoding abscisic acid 8′-hydroxylase (GLYMA09G35250) genes were up-regulated in *GmMAX3b-OE* hairy root. ABA synthesis genes, such as abscisic-aldehyde oxidase genes (GLYMA02G44000) were down-regulated in *GmMAX3b-OE* line, compared with the *GUS* control. ABSCISIC ACID-INSENSITIVE 5-like GmABI5 (GLYMA09G35250) was up-regulated by *GmMAX3b-OE* and down-regulated in *GmMAX3b*-*KD* hairy roots. For gibberellic acid (GA) biosynthesis and catabolism, gibberellin 2-beta-dioxygenase genes GLYMA15G10070, GLYMA13G33300, and GLYMA17G04150, and gibberellin 3-beta-dioxygenase genes GLYMA03G01190 and GLYMA13G0725, were up-regulated. Whereas gibberellin 20 oxidase genes GLYMA02G15390, GLYMA02G15380, and GLYMA02G15370 were down-regulated in *GmMAX3b-OE* hairy roots. Many genes encoding different types of transcription factors were up- or down-regulated in *GmMAX3b-OE* hairy roots, suggesting that they might be involved in mediation of SL-regulated downstream effects. TCP transcription factors BRCs were once been shown to act as down-stream effectors of SL signaling [2, 13]. In *GmMAX3b-OE* hairy roots, TCP genes such as GLYMA03G02090 and GLYMA09G42120 were markedly up-regulated as compared with that in *GUS* control. The C2H2 zinc-finger transcription factor STOP homolog genes, such as GLYMA18G02010 and GLYMA12G30285 were down-regulated in *GmMAX3b-OE*, suggesting that *GmMAX3b* or SL signaling also negatively regulates *GmSTOPs* and affects plant response to acidic soils and Al^3+^ stresses. In consistence, several aluminum-activated malate transporter (ALMT) family genes were also changed in *GmMAX3b*-OE hairy roots.

### *GmMAX3b-OE* and *GmMAX3b*-*KD* hairy roots altered the endogenous hormone levels

**Fig. 9:**
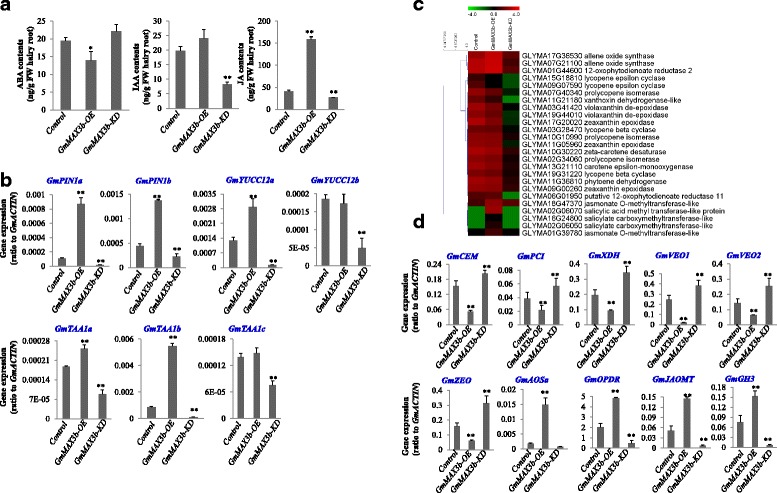
Effects of *GmMAX3b*-overexpression and *GmMAX3b* knockdown on hormone levels in hairy roots. Chimerical soybean plants with wild-type shoots and transgenic hairy roots overexpressing *GmMAX3b (GmMAX3b-OE), GmMAX3b*-knockdown (*GmMAX3b-KD)* and *GUS* vector control were inoculated with *Bradirhizobium japonicum* strain USDA110. Hairy roots were sampled for hormones and gene expression analysis at 28 days post inoculation. **a** Hormone contents in *GmMAX3b-OE, GmMAX3b-KD* and *GUS* hairy roots. **b** Validation of auxin biosynthetic genes *TAA*s and *YUC*s in *GmMAX3b-OE, GmMAX3b-KD* and *GUS* hairy root lines. **c** Heat map analysis of hormone biosynthesis and signaling genes in *GmMAX3b-OE, GmMAX3b-KD* and *GUS* hairy root lines. **d** Validation of SL biosynthesis and signaling genes in *GmMAX3b*-OE, *GmMAX3b-KD* and *GUS* hairy root lines. Data are expressed as means ± s.d from at least three independent experiments with duplicates. Differences are analyzed with student’s *t* test, *p < 0.05, **p < 0.01

Given various hormones had significant effects on legume nodulation processes, and GmMAX3b can affect the hormone levels in Arabidopsis *atmax3* mutant and wild-type plants, we thus examined hormone levels in the hairy roots of *GmMAX3b-OE, GmMAX3b*-*KD* and *GUS* chimerical soybean plants. It was found that *GmMAX3b-OE* hairy roots had a significantly increased while *GmMAX3b*-*KD* hairy roots had significantly decreased IAA level compared with *GUS* control (Fig. 9a). TRYPTOPHAN--PYRUVATE AMINOTRANSFERASE (TAA1) and flavone containing- proteins YUCCAs (YUC) have been shown to be critical for auxin biosynthesis [39, 69]. The expression of three major *TAA1*s, the major YUC genes in soybean roots, *GmYUC12a* (Glyma.03G208900.1) and *GmYUC12b* (Glyma.19G206200.1), as well as the putative *GmPIN* genes, *GmPIN1a* (Glyma.07G102500.1) and *GmPIN1b* (Glyma.08G054700.1), were examined in *GmMAX3b-OE, GmMAX3b*-*KD* and *GUS* hairy roots. *TAA1b* (Glyma17g09401), *GmYUC12a*, *GmPIN1a* and *GmPIN1b* were all expressed to higher levels in *GmMAX3b-OE* and lower in *GmMAX3b*-*KD* than in *GUS* control (Fig. 9b). More interestingly, the ABA level in *GmMAX3b-OE* hairy roots was significantly lower and higher in *GmMAX3b-KD* transgenic lines compared to the *GUS* control (Fig. 9a). JA contents were increased in *GmMAX3b-OE* and decreased in *GmMAX3b*-*KD* hairy roots compared to the *GUS* control (Fig. 9a). Examination of JA and ABA biosynthetic genes in GmMAX3b-OE, *GmMAX3b*-*KD* and *GUS* hairy roots lines confirmed that the altered expression of these genes revealed by RNA-seq data analyses. JA biosynthetic genes, *GmAOSa, GmOPDR, GmJAOMa*, as well as JA-specific GH3 gene *GmGH3a*, were up-regulated in *GmMAX3b-*OE and down-regulated in *GmMAX3b*-*KD* compared with *GUS* control hairy roots. Meanwhile, carotenoid pathway genes toward ABA biosynthesis, *GmCEMa, GmPCI, GmXDH, GmVEO1 GmVEO2*, and *GmZEO* were down-regulated in *GmMAX3b-OE* and up-regulated in *GmMAX3b*-*KD* hairy roots compared to the *GUS* control (Fig. 9c), supporting that ABA levels were decreased in *GmMAX3b-OE* and elevated in *GmMAX3b*-*KD* hairy roots.

### Subcellular localization of GmMAX3b

**Fig. 10:**
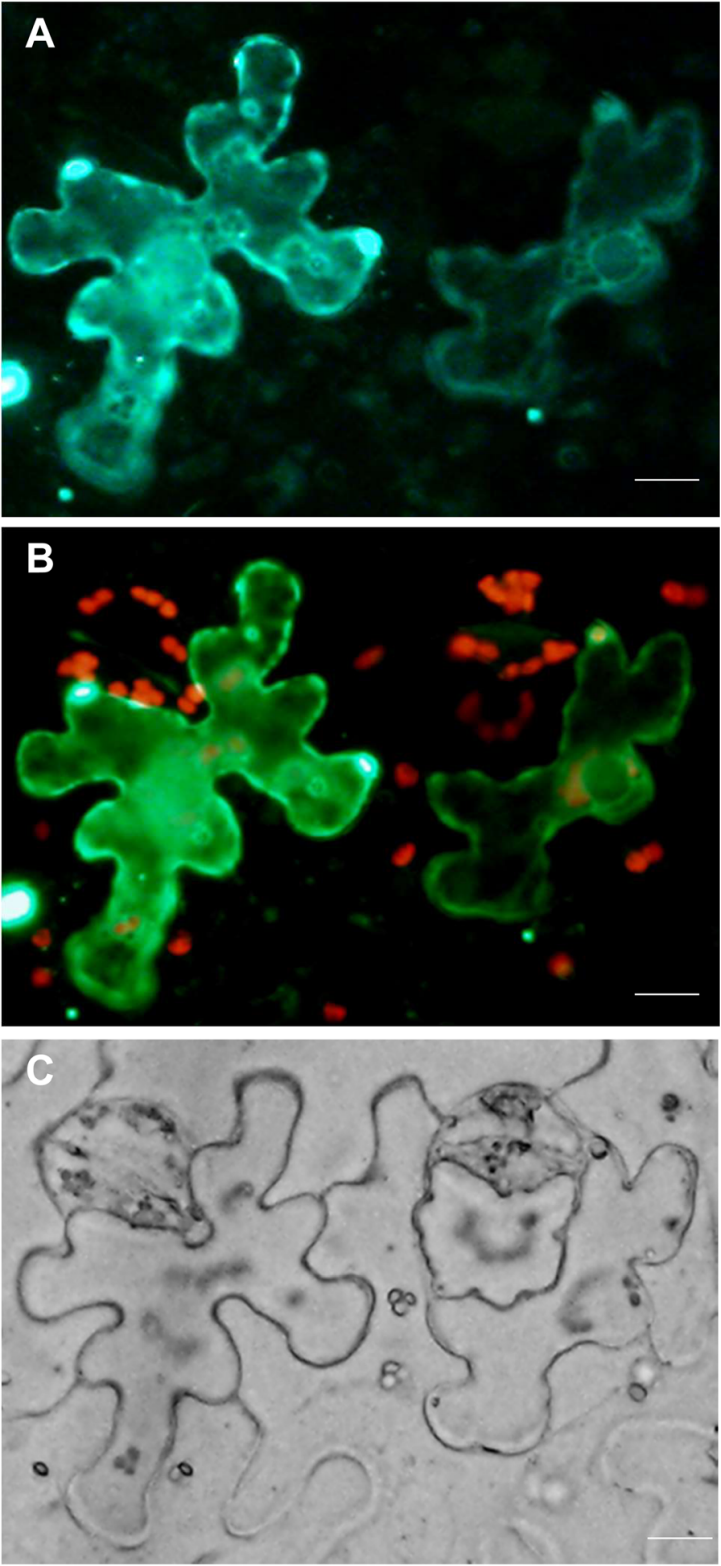
Subcellular localization of GFP-GmMAX3b. Transient expression of GFP- GmMAX3b fusion driven by a 35S promoter in tobacco leaf epidermal cells was observed under Olympus BX53 microscopy after 2 days of infiltration. **a** GFP-GmMAX3b image; **b** the merge of both GFP-GmMAX3b and chloroplast autofluorescence; **c** Bright field image. Bar = 30 μm

When GFP-GmMAX3b fusion was transiently expressed in the leaf epidermal cells of *Nicotiana benthamiana*, GFP-GmMAX3b signals was largely localized to chloroplasts and remained some in the cytosol (Fig. 10). This is consistent with the prediction that GmMAX3b protein has the first 30-aa chloroplast transit peptide for targeting to the chloroplasts by iPSORT (http://ipsort.hgc.jp/index.html). The signal of GFP-GmMAX3 in the cytosol may be due to the overexpression, which caused the many proteins not processed into the chloroplast (Fig. 10). This is in agreement with AtMAX3, which was also localized to the chloroplast in Arabidopsis.

## Discussion

As one of the most important economic crops, soybean becomes increasingly demanded for animal feeds, food industry, and sustainable agriculture for its nitrogen-fixation capability. As SLs play important roles in regulating plant architecture, shoot branching, root growth, plant-mycorrhizal and legume-rhizobium interaction, understanding of SLs in soybean are essential for soybean breading and agricultural practices. Unfortunately, so far SLs in soybean have not been systematically explored. The study tries to explore the unverified area to present a fresh scenario for the functions of SL biosynthesis and signaling genes in soybean in determining plant development and architecture and soybean nodulation.

### *GmMAXs* have conserved functions similar to their Arabidopsis counterparts

Unlike diploid model legumes *Medicago* or *Lotus* that usually contains only one copy of SLs biosynthesis gene homologues, tetraploid soybean genome owns multiple copies of each *MAX* or *D* gene. Many of them share high identity or similarity with pea, Arabidopsis and rice orthologs. As revealed in other plants, SLs usually are synthesized in roots and stem and transported upward to shoots and leaves [32, 73], SL biosynthesis genes show higher expression in the roots and stems, but SL signaling genes can be expressed everywhere. Indeed, the conserved SL biosynthesis and signaling components in soybean are also confirmed by expression of each *GmMAX* gene. Furthermore, ectopic expression of *GmMAX*s, *GmMAX1a, GmMAX2a, GmMAX3b,* and *GmMAX4a*, in Arabidopsis corresponding orthologous knockout mutants for genetic complementation and in wild-type Col-0 for overexpression, we observed the rescue of several typical phenotypes in the Arabidopsis mutants, or enhanced phenotypes by overexpression of corresponding *GmMAX* orthologs in the wild-type. *GmMAX1a, GmMAX2a, GmMAX3b,* and *GmMAX4a* could restore phenotypes of the *atmax* mutants in various tested aspects, including shoot height, shoot branching, leaf morphological development, root hair, and primary or lateral roots, to the wild-type (Figs. 2, 3, 4 and 5). All *atmax* mutants share some common phenotypes attributable to the lack of the SL biosynthesis and signaling, such as increased shoot branching, reduced height, decreased petiole length, leaf shape, and delayed leaf senescence (Figs. 2, 3, 4 and 5). Because AtMAX1, 3, and 4 are the biosynthesis enzymes, whereas AtMAX2 is a SL signaling component, different from *atmax1, atmax3*, and *atmax4* mutants, the *atmax2* mutants do not completely phenocopy *atmax1, atmax3,* and *atmax4* mutants [6, 16]. Although many *atmax1, 3,* and *4* mutant adult shoot phenotype are evident in the *atmax2–1* plant, the normal leaf blade length but wider leaf blade are observed in *atmax2*. AtMAX2 also has an additional role in both KAI2 signaling pathway, besides SL signaling pathway, thus *atmax2* has loss-of-functions in both SL- and KAI2 receptors-mediated signaling pathway [47, 67]. Additional phenotypes of *atmax2* mutants include the increased sensitivity to drought tolerance because of defects in cuticle development [16].

### *GmMAX3b* regulated hormone biosynthesis when expressed in Arabidopsis

Besides the phenotype complementation, *GmMAX*s also exerted effects on the biosynthesis of other hormones. *GmMAX*s rescued the abnormal hormone levels in these mutants, such as reduced auxin levels and increased JA and ABA contents, by overexpression of these *GmMAX*s. Overexpression of *GmMAX2a* in either *atmax2* mutant or in the wild-type could increase auxin levels. Overexpression of other *GmMAX*s, such as *GMAX1a, GmMAX3b,* and *GmMAX4a* repressed auxin biosynthesis, and rescued the increased IAA levels their orthologous mutants of Arabidopsis to the normal low levels (Fig. 7). However, all four *GmMAX*s could enhance JA and ABA contents in their Arabidopsis corresponding mutants, and the wild-type backgrounds.

It has been reported in a previously study that the SL synthesis mutants *atmax1–1*, *atmax3–9*, and *atmax4–1* have increased auxin transport in the primary inflorescence stem [8], and that *atmax1–1* and *atmax3–9* have increased levels of the PIN1 auxin efflux carrier at the basal plasma membrane of cambial and xylem parenchyma cells in the stem [6, 8]. Our results are consistent with these observations except for *atmax2* mutant, *atmax* mutant such as *atmax1, atmax3,* and *atmax4* plants all had significantly increased IAA levels. These increased levels of IAA, and enhanced transport of IAA caused more branches in these mutants. Expression of these *GmMAX*s in their corresponding mutants clearly restored the IAA levels to the normal (Fig. 7). All tested Arabidopsis *atmax* mutants had reduced levels of ABA and JA, which may be responsible for the delayed leaf senescence. Expression of these *GmMAX*s in their corresponding mutant backgrounds also enhanced the ABA and JA contents in these mutant backgrounds to the normal levels.

### *GmMAX3b* regulated soybean hairy root nodulation

Besides multiple physiological effects of SLs in root growth, shoot branching, and mycorrhizal branching, SLs have been implicated in legume nodulation [22–24]. The pea SLs-deficient mutant *rms1/CCD8* produces fewer nodules ~ 40% of the wild type, but application of synthetic SLs analog GR24 partially rescued the phenotype [22]. *Lotus japonicus LjCCD7*-silenced plants, showing 80% reduction in SLs, also reduced nodules by 20% compared with control plants [37, 38]. We also observed that overexpression and knockdown of *GmMAX3b* altered soybean nodulation. More than 26% increase in nodule number was detected in *GmMAX3b-OE* and 33% reduction was observed in *GmMAX3b*-*KD* hairy roots compared with *GUS* control, suggesting that GmMAX3b affected nodulation in chimerical transgenic hairy roots compared with *GUS* control. Nodulation gene expression confirmed that *GmMAX3b* overexpression and knockdown affected many key nodulation genes, like *NFR1α* genes involved in Nod factor perception, genes involved in Nod factor signal transduction such as *DMI2α*, and *DMI3β*, *NINα*, and *NSP2β*, to downstream factors, such as nodulin genes, *ENOD40, Rj4,* and *bHLH* [44, 45]. Transcriptome analysis also showed that overexpression of *GmMAX3b* down-regulated and *GmMAX3b* knockdown up-regulated several SL biosynthetic genes, such as *GmD27b, GmMAX1a, GmMAX4s*, and *GmMAX2s*, but slightly increased or decreased expression levels of *GmD14s* and *GmD53*. Thus overexpression and knockdown of *GmMAX3b* seems slightly down- or up-regulated SL biosynthesis and signaling. These data suggest that *GmMAX3b* affected SL biosynthesis and signaling, through which it may affect nodulation.

### *GmMAX3b* overexpression and knockdown changed expression of subsets of gene in soybean hairy roots

Our RNA-Seq data showed that more than 1077 genes were up-regulated and 1521 genes were down-regulated in *GmMAX3b-OE* hairy roots as compared with *GUS* control (Additional file 8: Data S1). Among these genes, some nodulation genes were up-regulated in GmMAX3b-OE and down-regulated in *GmMAX3b*-*KD* hairy roots, including several key nodulation genes. These regulation of genes were also confirmed by qRT-PCR. Therefore, the data supported that *GmMAX3b-OE* did promote soybean nodulation, by up-regulation of nodulation genes and *GmMAX3b-KD* decrease the soybean nodulation, by down-regulation of nodulation genes. In the further analysis of transcriptomic data, we did observe significantly increased expression levels of major auxin biosynthesis genes such as TAA1s, YUCCA1, 2, 4 in *GmMAX3b-OE* and vice versa in *GmMAX3b*-*KD* hairy roots. In Arabidopsis, *PIN* genes were regarded as one of major targets of SL downstream genes [6, 8]. Although it was shown that SLs regulate PIN1 through post-translational mechanism [6, 8], we had observed that several PIN genes were up-regulated in *GmMAX3b-OE* and down-regulated in *GmMAX3b*-*KD* hairy roots as compared with *GUS* lines. The up- or down-regulation of auxin signaling genes was consistent with slightly enhanced auxin levels in *GmMAX3b-OE* or decreased level in *GmMAX3b-KD* hairy roots than in *GUS* control lines. Obviously increased or decreased expression levels of jasmonate biosynthesis and metabolism genes, as well as JA downstream signaling genes in *GmMAX3b-OE* and *GmMAX3b*-*KD*, as supported by qRT-PCR analysis (Fig. 9), indicated that *GmMAX3b-OE* hairy roots had enhanced JA biosynthesis and signaling genes. This is exactly in line with our hormone analysis results, which showing that JA levels in *GmMAX3b-OE* are higher and lower in *GmMAX3b-KD* lines than these in *GUS* control. On the contrary, we see significantly decreased expression levels of many carotenoid genes, as well as ABA biosynthesis and signaling genes. These were consistent with qRT-PCR data, as well as the hormone analysis results, showing ABA contents were decreased in *GmMAX3b-OE*, but increased in *GmMAX3b-KD* hairy roots compared with *GUS* lines.

### *GmMAX3b* may modulate SL, auxin and other hormone signaling in soybean hairy roots to affect soybean nodulation

Auxins, JA, and ABA all impact mixed effects on SLs biosynthesis [15, 23]. SLs in turn affect these hormone biosynthesis and transport, and thereby regulate physiological processes [6, 8, 14, 61, 66]. We showed that *GmMAX3b-OE* hairy roots had slightly increased IAA levels, but drastically decreased ABA level, and significantly increased JA levels while *GmMAX3b-KD* hairy roots had significantly decreased IAA and JA levels, but slightly increased ABA level (Fig. 9a). MtD27 expression in nodulation not only depends on NSP1 and NSP2, but also is dependent of other symbiosis signaling pathway, including MtDMI1, MtDMI2, MtDMI3/MtCCaMK [62]. Pea SLs-deficient mutant *rms1/CCD8* showed defects in nodulation is dependent of SL signaling [22]. *Lotus japonicus LjCCD7*-silenced plants also showed defects on nodulation compared with control plants [37, 38]. Here we showed that *GmMAX3b-OE* caused alteration in SL biosynthesis and signaling genes, and most likely the SL effects. *GmMAX3b-OE* also caused slightly increased IAA levels and significantly increased JA levels, but decreased ABA levels, but *GmMAX3b*-*KD* had contrast hormone levels.

SL can either act as a suppressor or activator of JA and ABA biosynthesis, as complex hormone cross-talking networks. Jasmonic acid (JA) and ethylene could either negatively and positively regulate Nod factor signaling and nodulation process, depending on the different circumstances [41, 45, 56, 57]. ABA also can either suppress or support Nod factor signal transduction and nodule formation, depending on the ABA concentrations [21, 42, 58]. *GmMAX3b*-*OE* may promote the nodulation by decreasing ABA accumulation, increasing JA and IAA contents, both of them positively affect nodulation in soybean, whereas *GmMAX3b*-*KD* displayed opposite changes in these hormones (Fig. 8). Further investigations are needed to reveal how these hormones affect nodulation in soybean.

## Conclusion

In this study, an attempt was made to understand whether and how soybean SL biosynthesis and signaling are also suited for regulation of the relevant aspects of soybean plants. We identified the most closed homologues of AtMAX1/, AtMAX2/, AtMAX3/ and AtMAX4/ from soybean genome and further verified their capabilities on genetic complementation of Arabidopsis corresponding orthologs’ mutants. Not only morphological and developmental phenotypes were complemented by GmMAXs, the altered endogenous hormone levels were also complemented by overexpression of these GmMAX orhologs in these mutants. Further studies using GmMAX3b as an example, showed that GmMAX3b is involved in soybean root hair formation and nodulation, most likely through regulating various hormone levels in soybean hairy roots, as indicated by both transcriptomic profiling and hormone analyses. This study showed that SL modulates the level of other hormones and induces changes in plant development, including in soybean root hair developemnt and nodulation. GmMAX3b-medicated SL biosynthesis and signaling may affect soybean-rhizobia interaction, as indicated by altered early nodulation gene expression in GmMAX3b overexpression and knockdown mutant hairy roots.

## Additional files

Additional file 1: Table S1. List of primers used in this study. (DOC 84 kb)

Additional file 2: Figure S1. Amino acid sequence alignment and phylogenetic analyses of GmMAX1a. (PDF 778 kb)

Additional file 3: Figure S2. Amino acid sequence alignment and phylogenetic analyses of GmMAX2a. (PDF 772 kb)

Additional file 4: Figure S3. Amino acid sequence alignment and phylogenetic analyses of GmMAX3b. (PDF 981 kb)

Additional file 5: Figure S4. Amino acid sequence alignment and phylogenetic analyses of GmMAX4a. (PDF 719 kb)

Additional file 6: Figure S5. Expression patterns of SL biosynthesis and signaling genes in soybean. (PDF 809 kb)

Additional file 7: Figure S6. Semi qRT-PCR of *GmMAX1a, 2a, 3b and 4a* in Col-0, *max* mutants, complementation and overexpression Arabidopsis lines. (PDF 320 kb)

Additional file 8: Data S1. Transcriptomic expression of different genes in *GmMAX3b-OE* and *GmMAX3b-KD* hairy roots. (XLS 89 kb)

Additional file 9: Figure S7. Heat map analysis for the effects of *GmMAX3b* overexpression and knockdown on nodulation genes. (PDF 315 kb)

Additional file 10: Figure S8. Heat map analysis for the effects of *GmMAX3b* overexpression and knockdown on auxin biosynthesis and transport genes. (PDF 497 kb)

## Abbreviations

ABA: Abscisic acid
ABC: ATP-binding cassette
CCD: Carotenoid cleavage dioxygenase
D/HTD: Dwarf or high-tillering dwarf
D27: Dwarf27
DAD: Decreased apical dominance
DEGs: Differentially expressed genes
FPKM: Fragments per kilobase of transcript per million mapped reads
GA: Gibberellic acids
JA: Jasmonate acid
MAX: More axillary branching
MS: Murashige and Skoog
PDR1: Pleiotropic drug resistance1
qRT-PCR: Quantitative reverse transcriptase-PCR
RMS: Ramosus
SCF: SKP1–Cullin–F-Box
SLs: Strigolactones
SMXL6: SMAX1-Like6
