# Supplementary material for: Functional characterization of soybean strigolactone biosynthesis and signaling genes in Arabidopsis MAX mutants and GmMAX3 in soybean nodulation
Source: BMC Plant Biol. 2017 Dec 21;17:259. doi: 10.1186/s12870-017-1182-4 (PMC5740752; doi:10.1186/s12870-017-1182-4)
Supplement: Supplementary file 1 — List of primers used in this study. (DOC 84 kb) [file 12870_2017_1182_MOESM1_ESM.doc]

| **Gene**  Table S1. List of primers used in this study | **Primers** | **Purpose** |
| --- | --- | --- |
| **GmMAX1a**  (Glyma04g05510) | F:ATGGTGGTGTTCATGGATTATTTGGA  R:TTAGCAAGTCTCTGTTCTTTTTATG | For Cloning |
| **GmMAX1b**  (Glyma06g05520) | F: ATGGTATTCATGGATTATTTGGAATG  R: TTAGCAAGCCTCTTTTCTTTTTATG |
| **GmMAX2a**  (Glyma12g15360) | F: ATGGGCGACGGCAGCATCG  R: GTCAATCACAGATTCGACGCCTAT |
| **GmMAX3a**  (Glyma01g14266) | F: ATGCAAACCAAACCCATTCACAACA  R: CTAATTAGCTGCCCAGAAACCATGA |
| **GmMAX3b**  (Glyma11g16370) | F: ATGCAAGCCAAACCCATTCACAACA  R: CTAATTAGCTGCCCAGAAACCATG |
| **GmMAX4a**  (Glyma04g08910) | F: ATGGCTTTCACCACCTTGCCTTC  R: CTACTCTTTTGGAACCCAGCATC |
| **GmMAX4b**  (Glyma06g09000) | F: ATGGCTTTCACCTTCTCGCCT  R: TTACTCTTTTGGAACCCAGCATC |
| **qRT-GmMAX1a**  (GLYMA04G05510) | F: CCTCTCTAGTCCCCATTCAGTTT  R: TGTGTTGGTTGATGAAATCTGATAC | For qRT-PCR analyses |
| **qRT-GmMAX1b**  (GLYMA06G05520) | F: TACCGCAAATATTCTTCAGACC  R: ACCAGATTCTATCCCTTTCACC |
| **qRT-GmMAX1c**  (GLYMA17G34530) | F: TAGCCATGTTCACCACCCTTC  R: CAGGGCCGTATTTAGCCAGTA |
| **qRT-GmMAX2a**  (GLYMA12G15360) | F: TGTTTGCTCGCCAGGACTC  R: CACCATCACCCGGTTCATC |
| **qRT-GmMAX2b**  (GLYMA06G43000) | F: GGGTGTTCGAGACTGGTTAGAT  R: GTCAAGGTTTACGCAGCAAGA |
| **qRT-GmMAX3a**  (GLYMA01G14266) | F: CAGGCTACTCACCGTGTCTT  R: GGTATCCGTTACAGCCCAAT |
| **qRT-GmMAX3b**  (GLYMA11G16370) | F: AAACCCAAGTAAGAGCACATCC  R: CAACATGGAGCAACCACAACT |
| **qRT-GmMAX4a**  (GLYMA04G08910) | F: CTTTCACCACCTTGCCTTCA  R: TGGTTTCTTTCCGTTCCTCC |
| **qRT-GmMAX4b**  (GLYMA06G09000) | F: GAAATAAAGGCATCTACAAAGGGAA  R: ATGGTGGTGGTGGTGGCAGT |
| **qRT-GmD14a**  (Glyma17g35360) | F: CCTAGGTACAACAAATTCCTGAAC  R: AAGAGCGAGATGTCAGGGC |
| **qRT-GmD14b**  (Glyma0092s00240) | F: ACTACGAGGCCTGGGTGAAC  R: GGCCTCATGTTGAACAGCGT |
| **qRT-GmD27a**  (GLYMA02G16170) | F: ACTGATAAGGACACTGCTACCA  R: TGAGGTGAGAGAATCAGAGGT |
| **qRT-GmD27b**  (GLYMA10G03631) | F: TGTTTGCCGTCTTCACCA  R: TAAGAACCTTGGAAGCCGAC |
| **qRT-GmD27c**  (GLYMA13G10340) | F: CATCTGGTGAACCTGCTCCT  R: CCTGCTGGTGGATCTGAAAA |
| **qRT-GmD53a**  (GLYMA11G35410) | F: TTTGTGAGCGATGAAGAAGGT  R: TTCCCCACGAATTTCAAGTAAC |
| **qRT-GmD53b**  (GLYMA18G06990) | F: TCTTCATCACTTGTCAGACTCCC  R: ATCTACAGTTTCAAATCTCCCTTC |
| **qRT-GmPDR1a**  (GLYMA04G07420) | F: CAACCAAATGGCATCAGGAC  R: AACCACCACTTCTTCACATCAACT |
| **qRT-GmPDR1b**  (GLYMA06G07540) | F: TCTAGAGTTGATGTGAAGAAATGGT  R: TTGAATTAGGAGTAACATGTGACC |
| **qRT-GmPDR1c**  (GLYMA15G01490) | F: CTAAGTCAGCTTTTCATCATGGC  R: AATCTCAGCCAGTCCATTAAACA |
| **qRT-GmNFR1α**  (GLYMA02G43860) | F: ATTCACGAGCACACTGTGCCT  R: CCAAAATCTGCAACCTTTCC |
| **qRT-GmNFR5α**  (GLYMA01G38560) | F: TTCCCTTTCTTCCTCTCCAC  R: GCATGAAAAGTTTGTTCTATTGTC |
| **qRT-GmDMI2α**  (GLYMA01G02451 ) | F: GTCCTCAGTGGCCTTGACATT  R: ACACCCTTTTGCCTGCTTTG |
| **qRT-GmDMI3α**  (GLYMA15G35070) | F: AGTGTTTGGAGCACCGCAATC  R: TCAAACAAGTCAAATATACGTGGTG |
| **qRT-GmNSP1α**  (GLYMA16G01020) | F: CAACACTTATCTTCTTCTCCAACT  R: GGAAGCATTTGCTATGTTGTTAGG |
| **qRT-GmNSP2β**  (GLYMA07G04430) | F: AATCATTGCCAAGCGAAGCT  R: AGTCCAAAGCGAGGCAGAGA |
| **qRT-GmEnod40**  (Glyma01g03470.1) | F: GAAAGGGGTGTGAGAGGAGAG  R: CGCCACTCAAGAAAGAATGTT |
| **qRT-GmNINα**  (GLYMA04G00210) | F: TAACATGCGATGCTGATCTTG  R: TGATTTAGAGGCGAAGCTTGA |
| **qRT-GmPIN1a**  **(**GLYMA07G11550**)** | F: CTCAAATGGGTCCAAAAGTGTT  R: GAGACTGGTGAAGCACTTGAACT |
| **qRT-GmPIN1b**  **(**GLYMA08G05900**)** | F: GCTGGGGTTATGAGTGAATGAG  R: ATCCGTTGATCGATTCCTCCT |
| **qRT-GmTAA1a**  (GLYMA02G04270) | F: CTAACGAGTCATCTGAATCTTTC  R: ACCCTTTTCAGAAATTCATTGA |
| **qRT-GmTAA1b**  (GLYMA05G02521) | F: GGTAACGCTGTGACAGAAGGT  R: CCTTGCAAATTCGGTCTCTAA |
| **qRT-GmTAA1c**  ( GLYMA17G09401) | F: CAACTTGGATCATGGAGACCC  R: CCTCACTACTTCCCTTGCAAA |
| **qRT-GmYUC12a**  (Glyma03g36720) | F: GTACGACCAGGGCTGCCA  R: GGCTTTGAAATCCTTCCCAGAA | For qRT-PCR analyses |
| **qRT-GmYUC12b**  (Glyma19g39370) | F: GATGATGGATGGAAAGGGATT  R: CTCGTTCCCGTTTTTGTAGG |
| **qRT-GmCEM**  (Glyma.13G147500) | F: TAGTGGATAGGGTGTTTTGTAGATG  R: ACCAATAACATCAAGAGTCAACTGC |
| **qRT-GmPCI**  (Glyma.07G273600) | F:GAAGTGGTCAATTCATACGTGCTA  R:ACATTGAGGAGTCGTGTTAACCC |
| **qRT-GmXDH**  **(**Glyma.11G151700**)** | F:GACATCATAGTCAACAATGCTGGA  R:CACCTAAGGCACTTGCTACACTG |
| **qRT-GmVEO1**  **(**Glyma.03G253500**)** | F:CCAAATTAAGTGTGGGGACCT  R:AGGACATCAGGATTTGGAGCA |
| **qRT-GmVEO2**  **(**Glyma.19G251000**)** | F:TTCAGAAGTTGGCAGAAGGGT  R:CCTAATCGGCAAGGCACGT |
| **qRT-GmZEO**  **(**Glyma.17G174500**)** | F:GGTCCAAGGTGAGGAAGCAGT  R:TGTTTGTGTCCCAAGAATACTCG |
| **qRT-GmAOS1**  **(**Glyma.17G246500**)** | F: TCGCCACCTGCTTTAACTCC  R: TCATCAGCGGCATCTTCTCC |
| **qRT-GmAOS2**  **(**Glyma.07G162900**)** | F: TTGGAGCAATAAGCGACAGAC  R: GGGTCCGAGGAGATGAAGG |
| **qRT-GmOPDR**  **(**Glyma.01G235600**)** | F: AGATAAAGTGAATGACCGAACAGAC  R: GCGATAGCCTAATTCCAACTCTG |
| **qRT-GmJAOMT**  **(**Glyma.18G238800**)** | F: GAATGATGGCAATAAAGAACAGAG  R: AGTGTATTTGGTCCCGCAGAG |
| **qRT-GmIAAaS**  **(**Glyma.01G190600**)** | F: GGTCTTTGCCGTTACAGGGT  R: TCAGCGAAGCTTGTGTACTCC |
| **qRT-GmABI5**  **(**Glyma.19G122800**)** | F: AGGGAGCCTAACATTGAGTGG  R: CAAGAAATCCTCCAGGGTCATC |
| **qRT-GmACTIN** | F: CTTCCCTCAGCACCTTCCAA  R: GGTCCAGCTTTCACACTCCAT |
