# Supplementary material for: Functional characterization of soybean strigolactone biosynthesis and signaling genes in Arabidopsis MAX mutants and GmMAX3 in soybean nodulation
Source: BMC Plant Biol. 2017 Dec 21;17:259. doi: 10.1186/s12870-017-1182-4 (PMC5740752; doi:10.1186/s12870-017-1182-4)
Supplement: Supplementary file 2 — Amino acid sequence alignment and phylogenetic analyses of GmMAX1a. (PDF 778 kb) [file 12870_2017_1182_MOESM2_ESM.pdf]

A

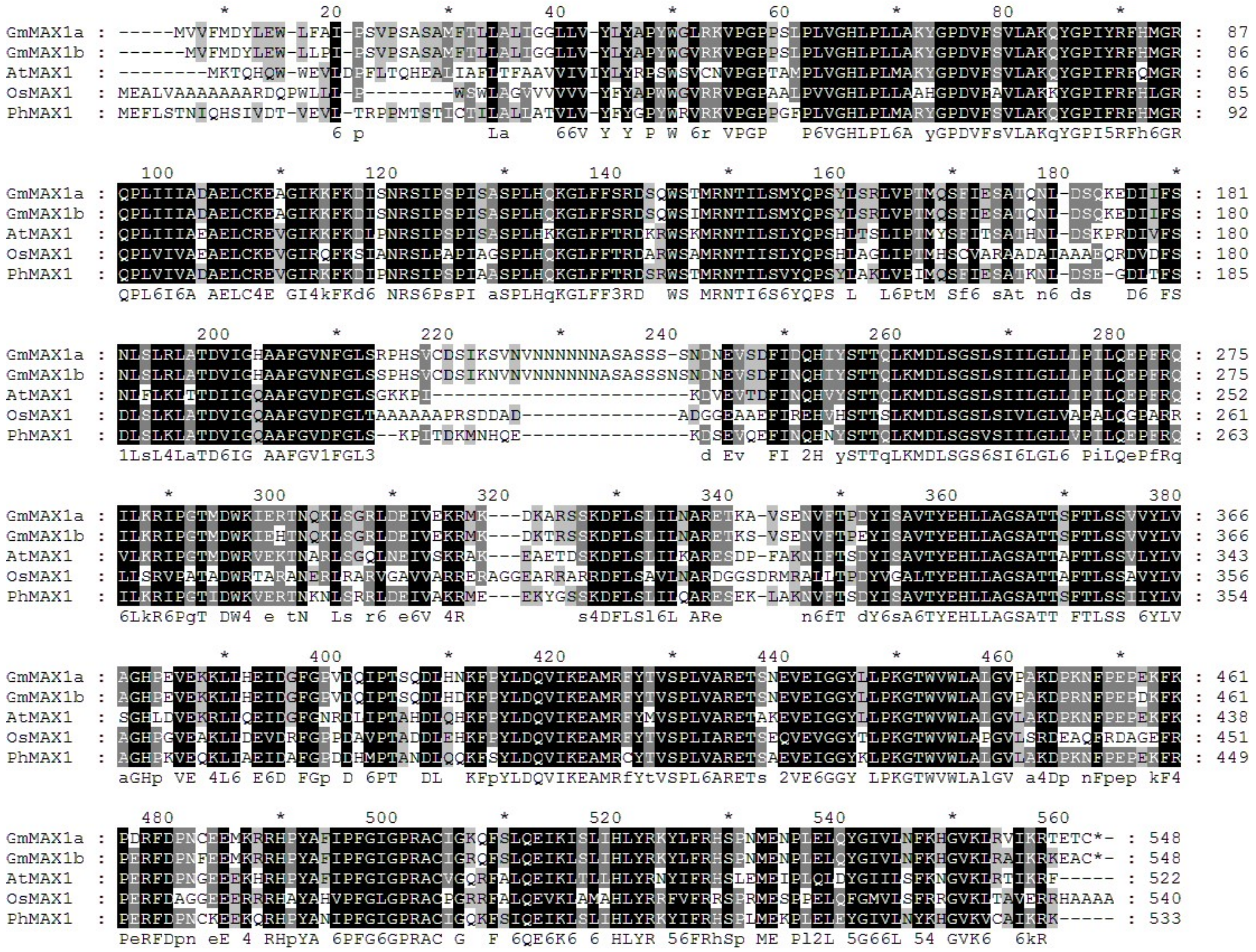
GmMAX1a
GmMAX1b
AtMAX1
OsMAX1
PhMAX1

B

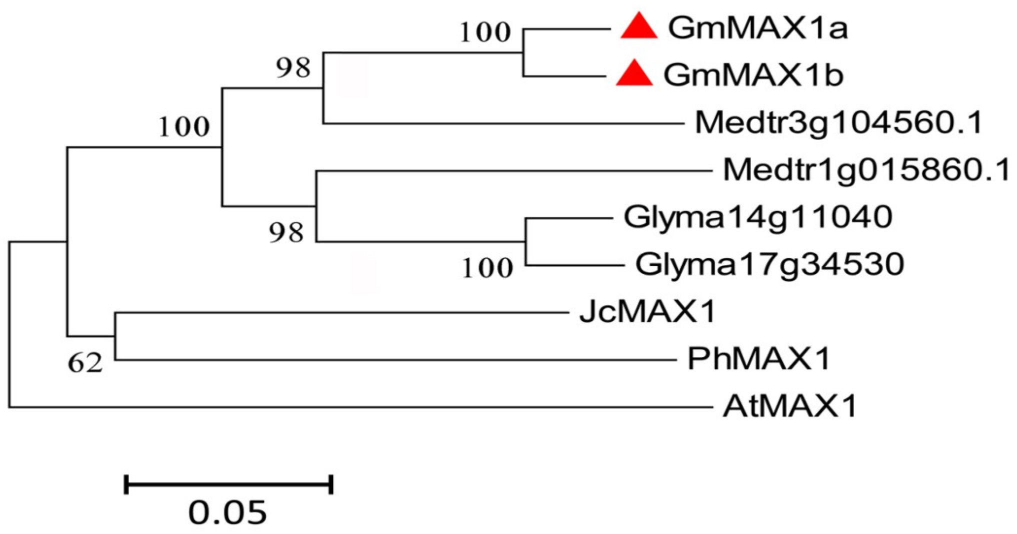
GmMAX1a
GmMAX1b
Medtr3g104560.1
Medtr1g015860.1
Glyma14g11040
Glyma17g34530
JcMAX1
PhMAX1
AtMAX1
100
98
100
98
100
62
0.05

Figure S1. Amino acid sequence alignment and phylogenetic analyses of GmMAX1a

(**A)** Amino acid sequence alignment of GmMAX1a with GmMAX1b, PhMAX1 and OsMAX1. MEGA6 was used for the alignment of GmMAX1a (used in this study) with AtMAX1 (B9DFU2), PhMAX1 (AEB97383.1) and OsMAX1 (AGI65364). MEGA6 alignment was used in GeneDoc program to shade the indistinguishable and similar amino acids in alignment. Dark shade represents identical amino acids and grey shade designate similar amino acids among genes and Dashes lines designate gaps in the alignment.

(B) Phylogenetic analysis of SL biosynthesis and signaling genes.

phylogenetic tree was constructed using soybean SL proteins with other functionally Characterized SL genes from Arabidopsis, Medicago, Pea, Petunia and rice with MEGA6 program through neighbor joining method. The bootstrap values were based on 1000 replicates.
