# Supplementary material for: Functional characterization of soybean strigolactone biosynthesis and signaling genes in Arabidopsis MAX mutants and GmMAX3 in soybean nodulation
Source: BMC Plant Biol. 2017 Dec 21;17:259. doi: 10.1186/s12870-017-1182-4 (PMC5740752; doi:10.1186/s12870-017-1182-4)
Supplement: Supplementary file 3 — Amino acid sequence alignment and phylogenetic analyses of GmMAX2a. (PDF 772 kb) [file 12870_2017_1182_MOESM3_ESM.pdf]

A

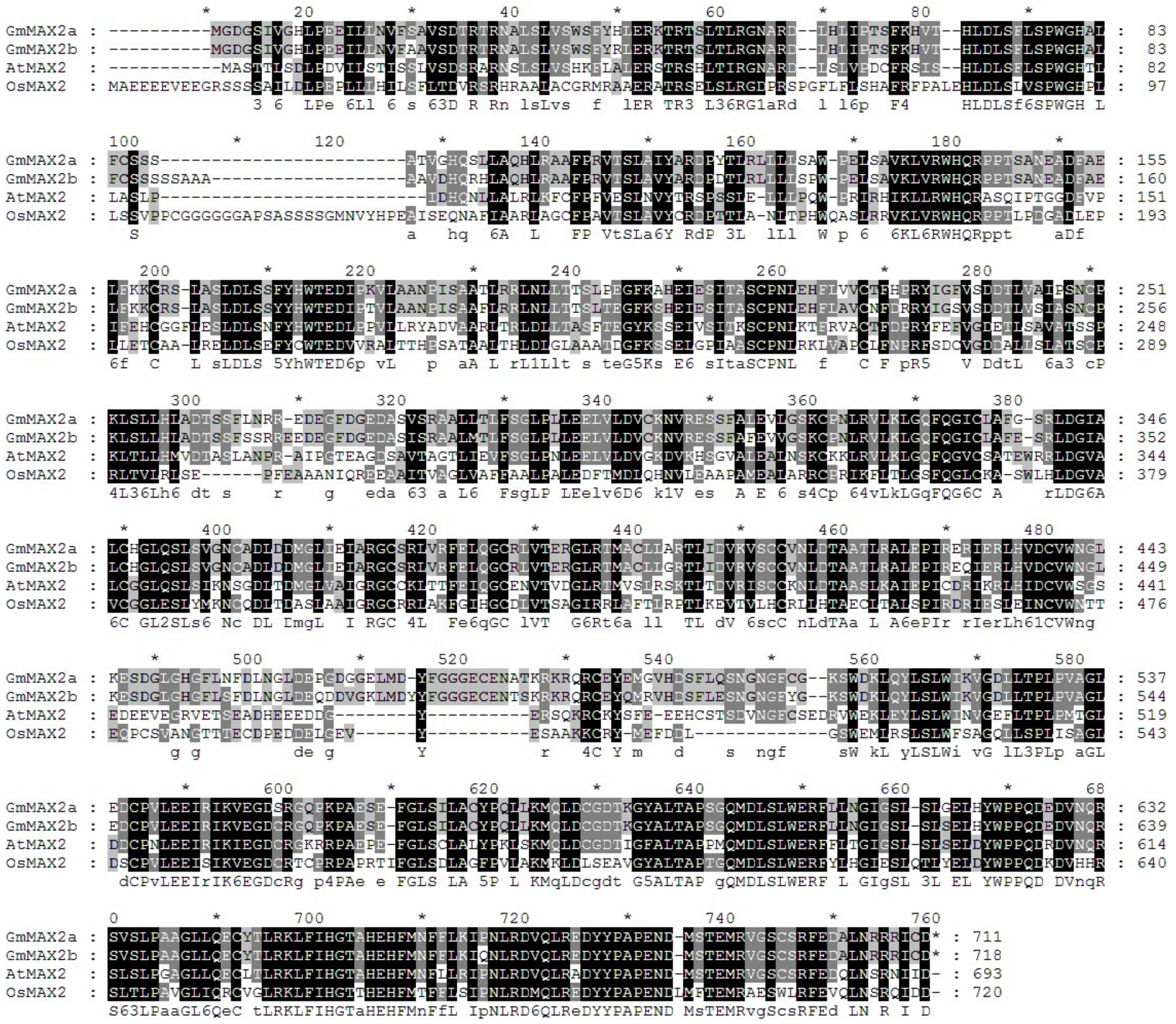
GmMAX2a
GmMAX2b
AtMAX2
OsMAX2

B

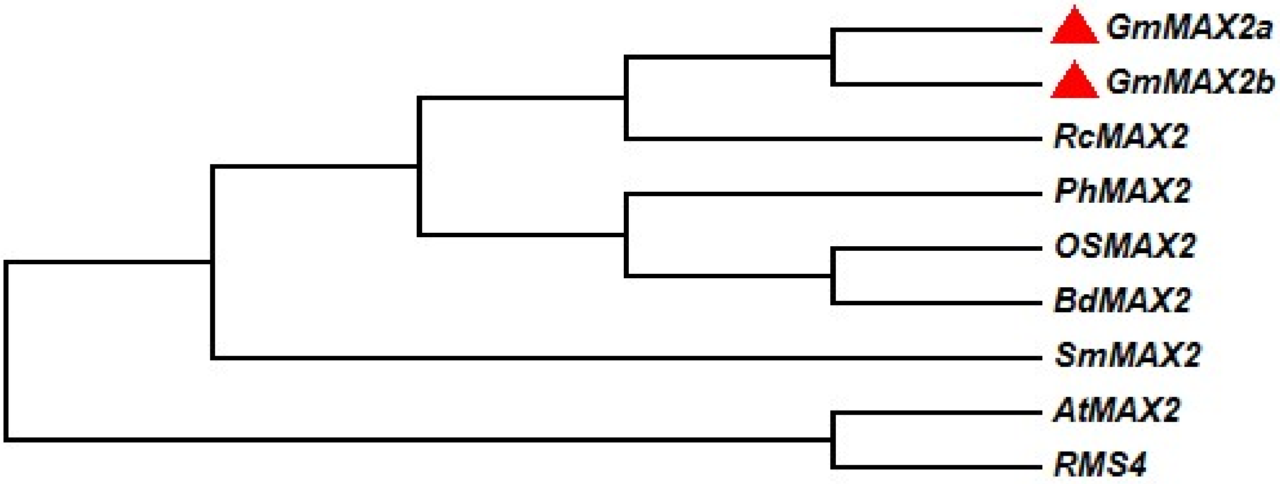
GmMAX2a
GmMAX2b
RcMAX2
PhMAX2
OSMAX2
BdMAX2
SmMAX2
AtMAX2
RMS4

Figure S2. Amino acid sequence alignment and phylogenetic analyses of GmMAX2a

(**A)**Amino acid sequence alignment of GmMAX2a with GmMAX2b, AtMAX2 and OsMAX2. MEGA6 was used for the alignment of GmMAX2a (used in this study) with AtMAX2 (Q9SIM9.2) and OsMAX2 (Q5VMP0.2). MEGA6 alignment was used in GeneDoc program to shade the identical and similar amino acids in alignment. Dark shade represents identical amino acids and grey shade indicates similar amino acids among genes

(B) Phylogenetic analysis of SL biosynthesis and signaling genes.

phylogenetic trees was constructed using soybean SL proteins with other functionally Characterized SL genes from Arabidopsis, Medicago, Pea, Petunia and rice with MEGA6 program through neighbor joining method. The bootstrap values were based on 1000 replicates.
