# Supplementary material for: Functional characterization of soybean strigolactone biosynthesis and signaling genes in Arabidopsis MAX mutants and GmMAX3 in soybean nodulation
Source: BMC Plant Biol. 2017 Dec 21;17:259. doi: 10.1186/s12870-017-1182-4 (PMC5740752; doi:10.1186/s12870-017-1182-4)
Supplement: Supplementary file 4 — Amino acid sequence alignment and phylogenetic analyses of GmMAX3b. (PDF 981 kb) [file 12870_2017_1182_MOESM4_ESM.pdf]

A

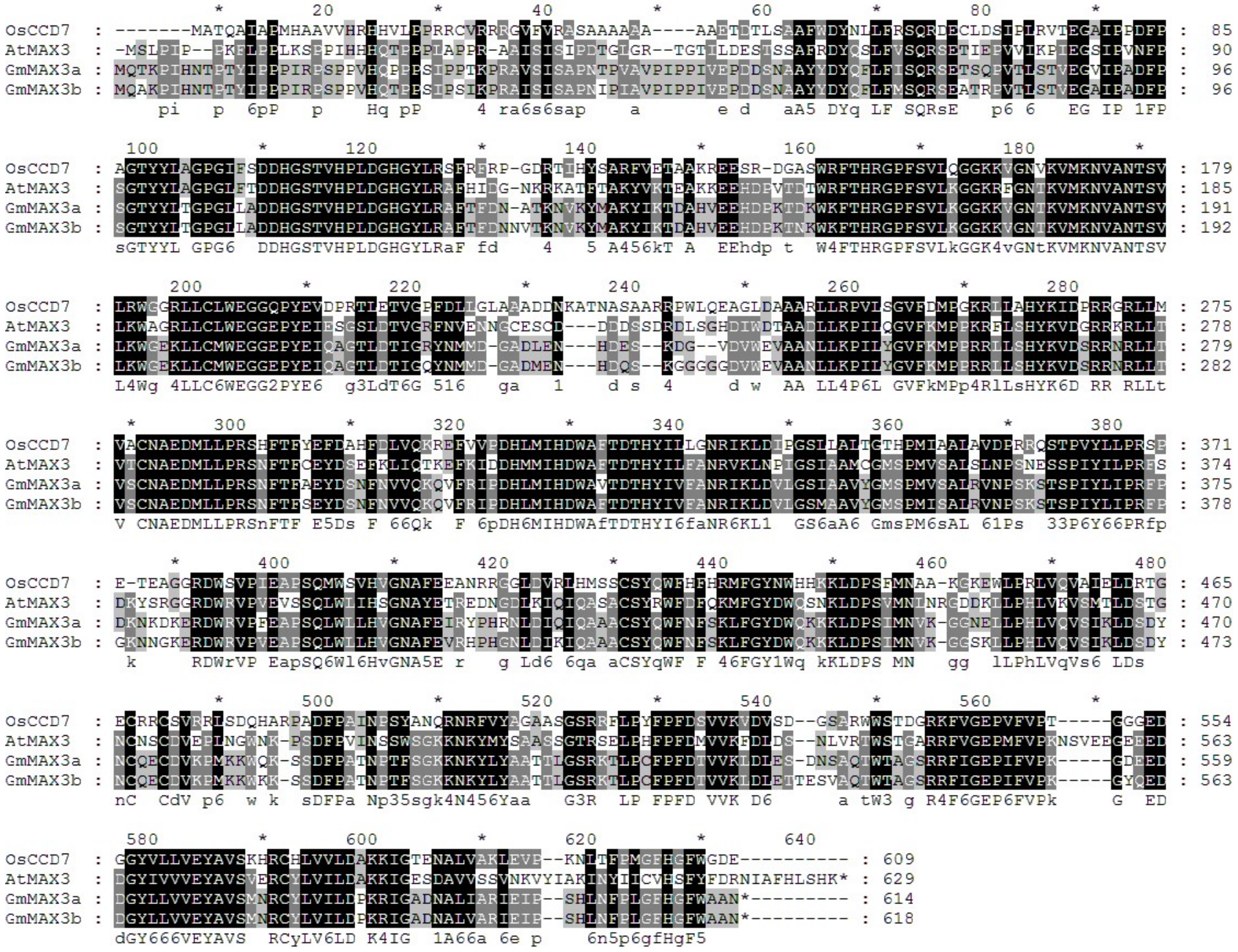
OsCCD7
AtMAX3
GmMAX3a
GmMAX3b

B

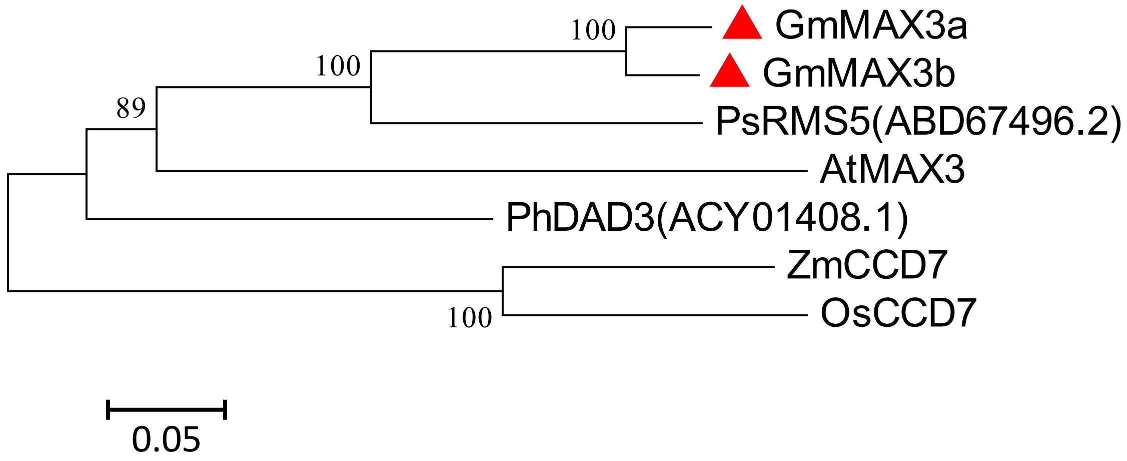
GmMAX3a
GmMAX3b
PsRMS5(ABD67496.2)
AtMAX3
PhDAD3(ACY01408.1)
ZmCCD7
OsCCD7
100
100
89
100
0.05

Figure S3. Amino acid sequence alignment and phylogenetic analyses of GmMAX3b

(A) Amino acid sequence alignment of *GmMAX3a* with GmMAX3b, AtMAX3 and OsMAX3. MEGA6 was used for the alignment of GmMAX3b (used in this study, G. max) with AtMAX3 (Q7XJM2) and OsMAX3 (Q7XU29). MEGA6 alignment was used in GeneDoc program to shade the identical and similar amino acids in alignment. Dark shade represents identical amino acids and grey shade indicates similar amino acids among genes and Dashes lines designate gaps in the alignment.

(B) Phylogenetic analysis of SL biosynthesis and signaling genes.

Phylogenetic tree was constructed using soybean SL proteins with other functionally characterized SL genes from Arabidopsis, Medicago, Pea, Petunia and rice with MEGA6 program through neighbor joining method. The bootstrap values were based on 1000 replicates.
