# Supplementary material for: Functional characterization of soybean strigolactone biosynthesis and signaling genes in Arabidopsis MAX mutants and GmMAX3 in soybean nodulation
Source: BMC Plant Biol. 2017 Dec 21;17:259. doi: 10.1186/s12870-017-1182-4 (PMC5740752; doi:10.1186/s12870-017-1182-4)
Supplement: Supplementary file 5 — Amino acid sequence alignment and phylogenetic analyses of GmMAX4a. (PDF 719 kb) [file 12870_2017_1182_MOESM5_ESM.pdf]

A

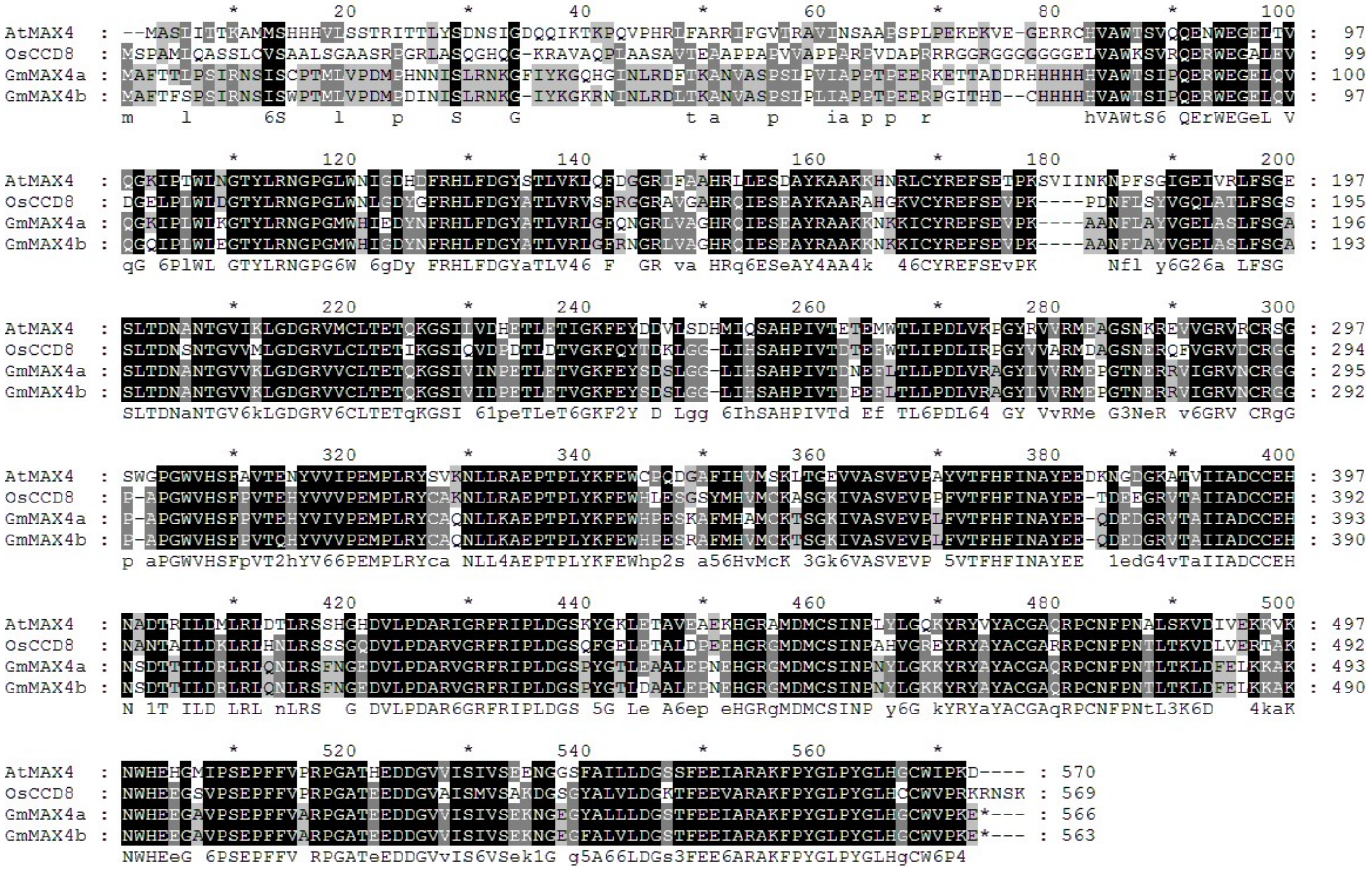
AtMAX4
OsCCD8
GmMAX4a
GmMAX4b

B

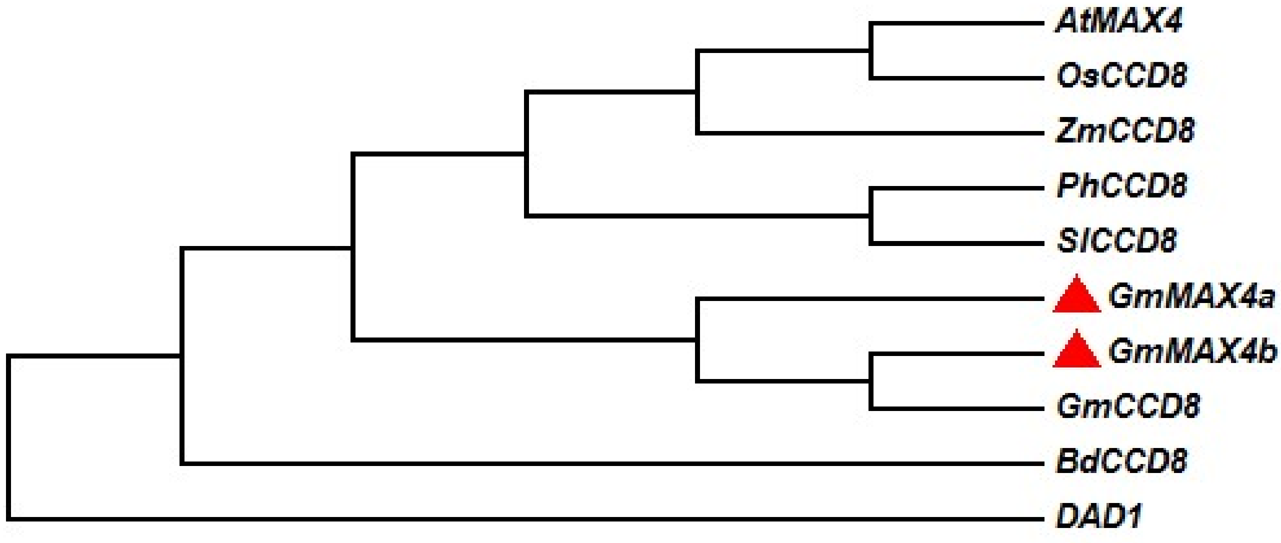
AtMAX4
OsCCD8
ZmCCD8
PhCCD8
SlCCD8
GmMAX4a
GmMAX4b
GmCCD8
BdCCD8
DAD1

Figure S4. Amino acid sequence alignment and phylogenetic analyses of GmMAX4a

(A) Amino acid sequence alignment of *GmMAX4a* with GmMAX4b, AtMAX4 and OsMAX4. MEGA6alignment of GmMAX4a (used in this study) with AtMAX4 (Q8VY26) and OsMAX4 (Q93VD5). MEGA6 alignment was used in Gene Doc program to shade the identical and similar amino acids in alignment. Dark shade represents identical amino acids and grey shade indicates similar amino acids among genes and Dashes lines designate gaps in the alignment.

(B) Phylogenetic analysis of SL biosynthesis and signaling genes.

Phylogenetic tree was constructed using soybean SL proteins with other functionally Characterized SLs genes from Arabidopsis, Medicago, Pea, Petunia and rice, maize and tomato with MEGA6 program through neighbor joining method. The bootstrap values were based on 1000 replicates.
