# Supplementary material for: Functional characterization of soybean strigolactone biosynthesis and signaling genes in Arabidopsis MAX mutants and GmMAX3 in soybean nodulation
Source: BMC Plant Biol. 2017 Dec 21;17:259. doi: 10.1186/s12870-017-1182-4 (PMC5740752; doi:10.1186/s12870-017-1182-4)
Supplement: Supplementary file 6 — Expression patterns of SL biosynthesis and signaling genes in soybean. (PDF 809 kb) [file 12870_2017_1182_MOESM6_ESM.pdf]

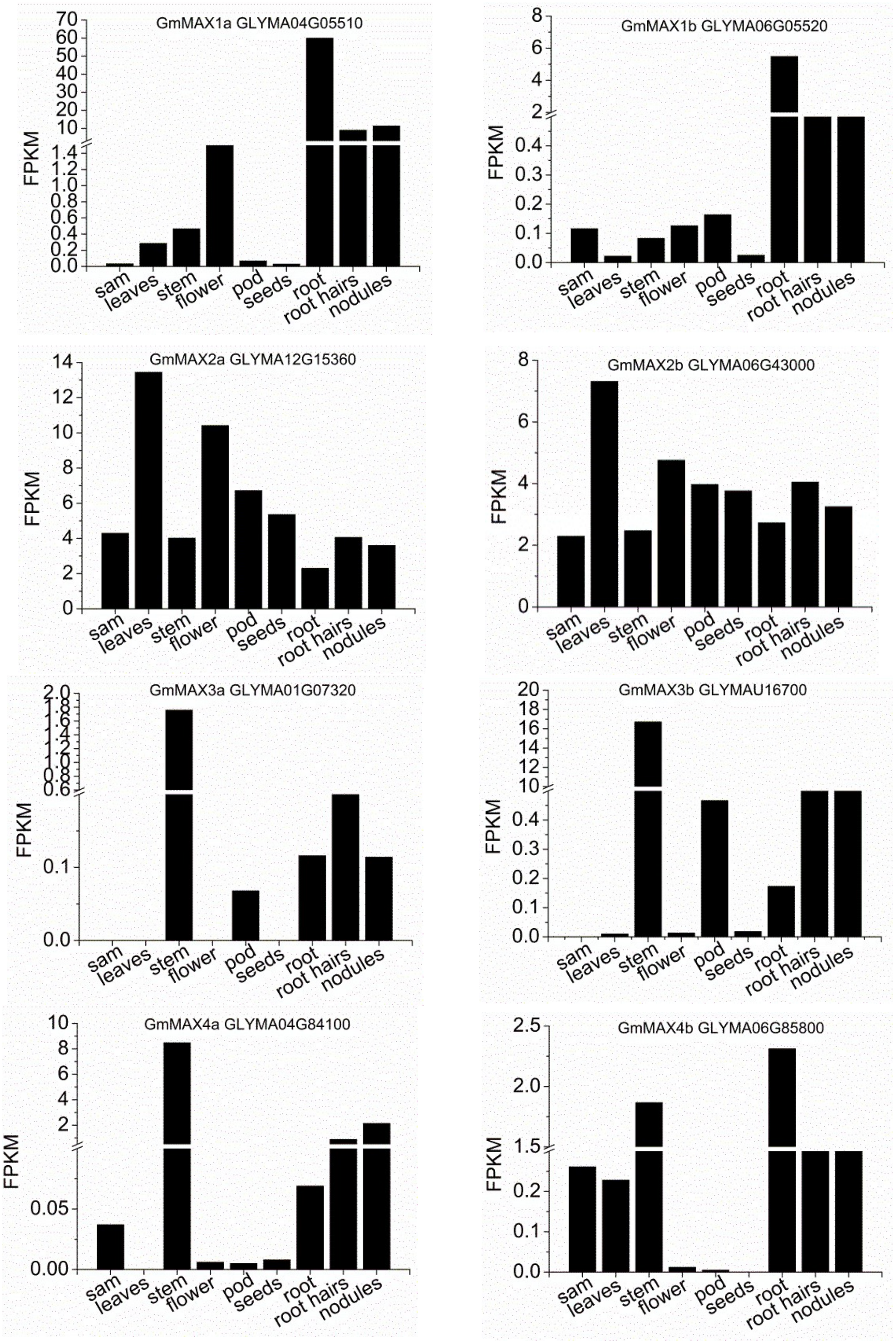

Figure S5. Expression patterns of SL biosynthesis and signaling genes in soybean

The public data of GmMAX1a (GLYMA04G05510), GmMAX1b (GLYMA06G05520), GmMAX2a (GLYMA12G15360), GmMAX2b (GLYMA10G03631), GmMAX3a (Glyma01g07320), GmMAX3b (GlymaU16700), GmMAX4a (Glyma04g84100), GmMAX4b (Glyma06g85800) are retrieived from phytozome (https://phytozome.jgi.doe.gov/).
