# Supplementary material for: Functional characterization of soybean strigolactone biosynthesis and signaling genes in Arabidopsis MAX mutants and GmMAX3 in soybean nodulation
Source: BMC Plant Biol. 2017 Dec 21;17:259. doi: 10.1186/s12870-017-1182-4 (PMC5740752; doi:10.1186/s12870-017-1182-4)
Supplement: Supplementary file 7 — Semi qRT-PCR of GmMAX1a, 2a, 3b and 4a in Col-0, max mutants, complementation and overexpression Arabidopsis lines. (PDF 320 kb) [file 12870_2017_1182_MOESM7_ESM.pdf]

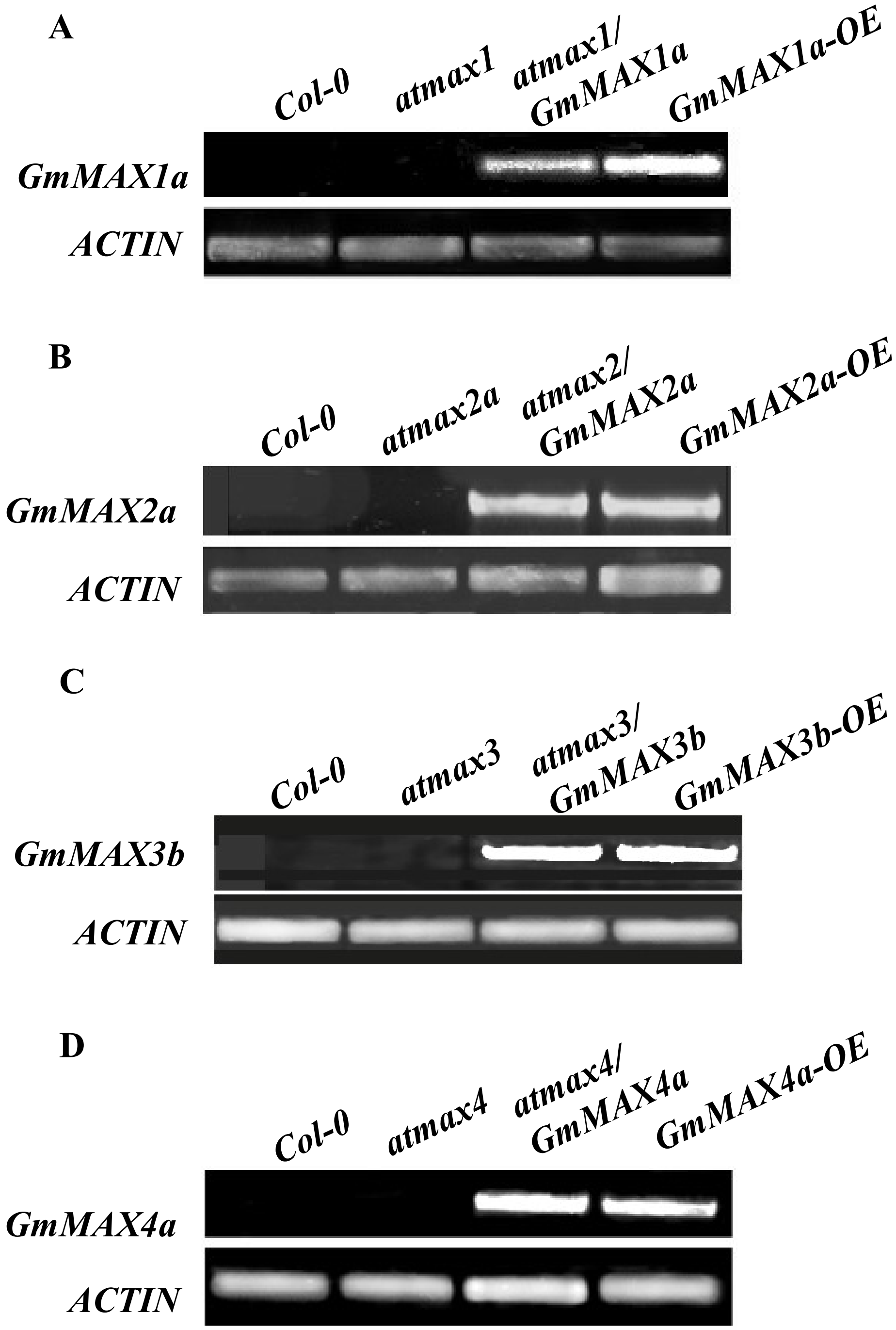

Figure S6. Semi qRT-PCR of GmMAX1a, 2a, 3b and 4a in Col-0, mutant, complement and overexpressed Arabidopsis lines. Leaf were taken from Arabidopsis lines, grinded in powder, RNA was extracted and PCR was done with respective primers. ACTIN was used as internal control.
