# Supplementary material for: Functional characterization of soybean strigolactone biosynthesis and signaling genes in Arabidopsis MAX mutants and GmMAX3 in soybean nodulation
Source: BMC Plant Biol. 2017 Dec 21;17:259. doi: 10.1186/s12870-017-1182-4 (PMC5740752; doi:10.1186/s12870-017-1182-4)
Supplement: Supplementary file 9 — Heat map analysis for the effects of GmMAX3b overexpression and knockdown on nodulation genes. (PDF 315 kb) [file 12870_2017_1182_MOESM9_ESM.pdf]

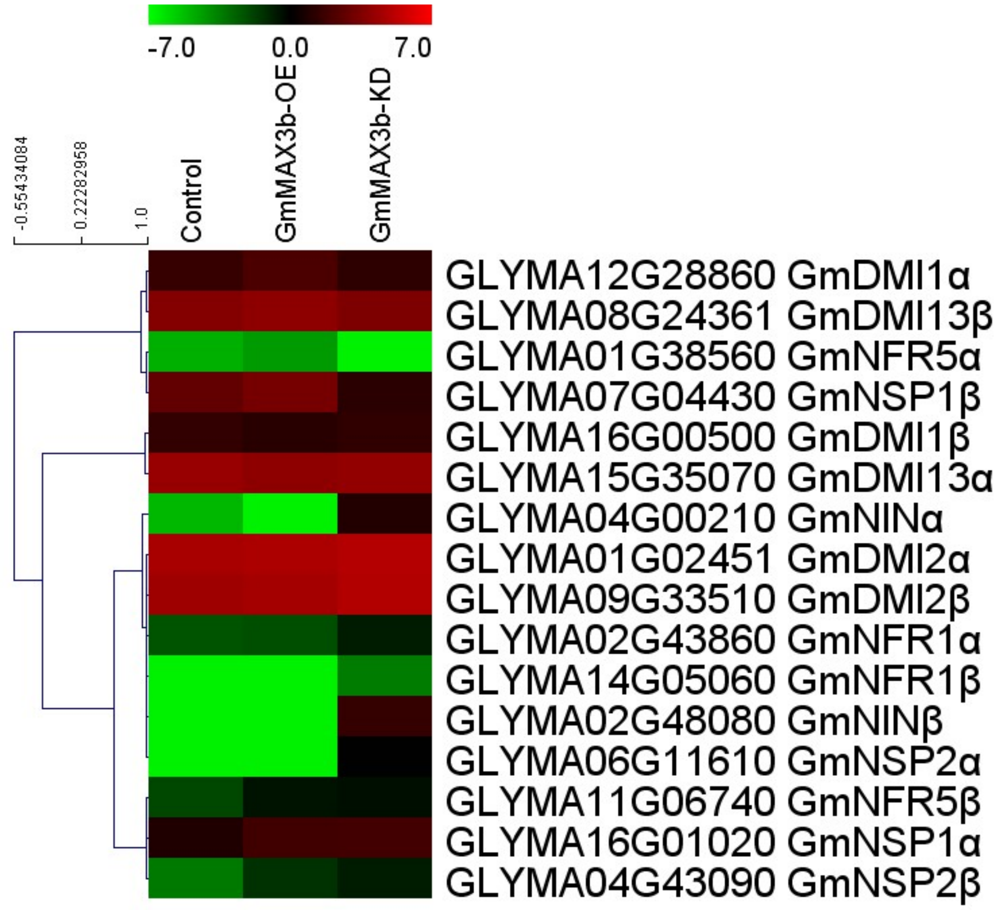

Figure S7. Heat map for the effects of GmMAX3b overexpression on nodulation . Heat map analysis of nodulation gene expression in GmMAX3b-OE hairy roots were done with program ((MeVv4.8 software (http://www.tm4.org/) in comparison with the GUS control, based on transcriptome data
