# Supplementary material for: Functional characterization of soybean strigolactone biosynthesis and signaling genes in Arabidopsis MAX mutants and GmMAX3 in soybean nodulation
Source: BMC Plant Biol. 2017 Dec 21;17:259. doi: 10.1186/s12870-017-1182-4 (PMC5740752; doi:10.1186/s12870-017-1182-4)
Supplement: Supplementary file 10 — Heat map analysis for the effects of GmMAX3b overexpression and knockdown on auxin biosynthesis and transport genes. (PDF 497 kb) [file 12870_2017_1182_MOESM10_ESM.pdf]

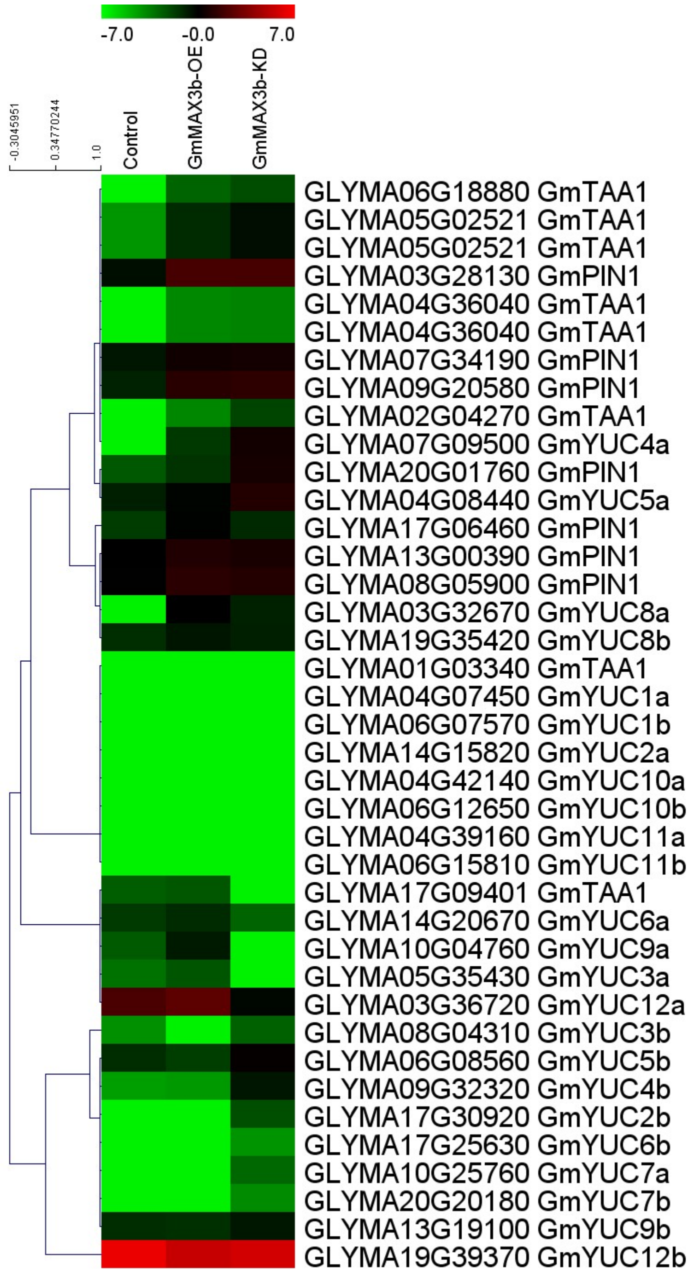


**Figure S8.** Heat map for the effects of GmMAX3b overexpression on auxin biosynthesis and transport genes. The heat map analysis of auxin biosynthetic genes such as TAA and YUCCA gene expression in GmMAX3b-OE hairy roots were done with program (MeVv4.8 software (http://www.tm4.org/) in comparison with the GUS control, based on transcriptome data
